# Signalling by senescent melanocytes hyperactivates hair growth

**DOI:** 10.1038/s41586-023-06172-8

**Published:** 2023-06-21

**Authors:** Xiaojie Wang, Raul Ramos, Anne Q. Phan, Kosuke Yamaga, Jessica L. Flesher, Shan Jiang, Ji Won Oh, Suoqin Jin, Sohail Jahid, Chen-Hsiang Kuan, Truman Kt Nguyen, Heidi Y. Liang, Nitish Udupi Shettigar, Renzhi Hou, Kevin H. Tran, Andrew Nguyen, Kimberly N. Vu, Jennie L. Phung, Jonard P. Ingal, Katelyn M. Levitt, Xiaoling Cao, Yingzi Liu, Zhili Deng, Nobuhiko Taguchi, Vanessa M. Scarfone, Guangfang Wang, Kara Nicole Paolilli, Xiaoyang Wang, Christian F. Guerrero-Juarez, Ryan T. Davis, Elyse Noelani Greenberg, Rolando Ruiz-Vega, Priya Vasudeva, Rabi Murad, Lily Halida Putri Widyastuti, Hye-Lim Lee, Kevin J. McElwee, Alain-Pierre Gadeau, Devon A. Lawson, Bogi Andersen, Ali Mortazavi, Zhengquan Yu, Qing Nie, Takahiro Kunisada, Michael Karin, Jan Tuckermann, Jeffrey D. Esko, Anand K. Ganesan, Ji Li, Maksim V. Plikus

**Affiliations:** 1 Department of Developmental and Cell Biology, University of California, Irvine, CA USA; 2 Sue and Bill Gross Stem Cell Research Center, University of California, Irvine, CA USA; 3 NSF-Simons Center for Multiscale Cell Fate Research, University of California, Irvine, CA USA; 4 Glycobiology Research and Training Center, Department of Cellular and Molecular Medicine, University of California, San Diego, La Jolla, CA USA; 5 Department of Biological Chemistry, University of California, Irvine, CA USA; 6 Center for Complex Biological Systems, University of California, Irvine, CA USA; 7 Department of Anatomy, Yonsei University College of Medicine, Seoul, Korea; 8 Department of Anatomy and Hair Transplantation Center, Kyungpook National University and Hospital, Daegu, Korea; 9 Department of Mathematics, University of California, Irvine, CA USA; 10 School of Mathematics and Statistics, Wuhan University, Wuhan, China; 11 Division of Plastic Surgery, Department of Surgery, National Taiwan University Hospital, Taipei, Taiwan; 12 Research Center for Developmental Biology and Regenerative Medicine, National Taiwan University, Taipei, Taiwan; 13 Amplifica Holdings Group, Inc., San Diego, CA USA; 14 Department of Dermatology, Xiangya Hospital, Central South University, Changsha, China; 15 Department of Tissue and Organ Development, Regeneration and Advanced Medical Science, Gifu University Graduate School of Medicine, Gifu, Japan; 16 Department of Physiology and Biophysics, University of California, Irvine, CA USA; 17 Centre for Skin Sciences, University of Bradford, Bradford, UK; 18 University of Bordeaux, INSERM U1034, Adaptation cardiovasculaire à l’ischémie, Pessac, France; 19 Department of Medicine, University of California, Irvine, CA USA; 20 State Key Laboratory of Farm Animal Biotech Breeding, College of Biological Sciences, China Agricultural University, Beijing, China; 21 Laboratory of Gene Regulation and Signal Transduction, Departments of Pharmacology and Pathology, University of California San Diego, School of Medicine, La Jolla, CA USA; 22 Institute for Comparative Molecular Endocrinology (CME), University of Ulm, Helmholtzstrasse 8/1, Ulm, Germany; 23 Leibniz Institute on Aging-Fritz Lipmann Institute, Beutenbergstrasse 11, Jena, Germany; 24 Department of Dermatology, University of California, Irvine, CA USA; 25 Hunan Key Laboratory of Aging Biology, Xiangya Hospital, Central South University, Changsha, China

**Keywords:** Senescence, Skin stem cells, Stem-cell niche, Extracellular signalling molecules

## Abstract

Niche signals maintain stem cells in a prolonged quiescence or transiently activate them for proper regeneration^1^. Altering balanced niche signalling can lead to regenerative disorders. Melanocytic skin nevi in human often display excessive hair growth, suggesting hair stem cell hyperactivity. Here, using genetic mouse models of nevi^2,3^, we show that dermal clusters of senescent melanocytes drive epithelial hair stem cells to exit quiescence and change their transcriptome and composition, potently enhancing hair renewal. Nevus melanocytes activate a distinct secretome, enriched for signalling factors. Osteopontin, the leading nevus signalling factor, is both necessary and sufficient to induce hair growth. Injection of osteopontin or its genetic overexpression is sufficient to induce robust hair growth in mice, whereas germline and conditional deletions of either osteopontin or CD44, its cognate receptor on epithelial hair cells, rescue enhanced hair growth induced by dermal nevus melanocytes. Osteopontin is overexpressed in human hairy nevi, and it stimulates new growth of human hair follicles. Although broad accumulation of senescent cells, such as upon ageing or genotoxic stress, is detrimental for the regenerative capacity of tissue^4^, we show that signalling by senescent cell clusters can potently enhance the activity of adjacent intact stem cells and stimulate tissue renewal. This finding identifies senescent cells and their secretome as an attractive therapeutic target in regenerative disorders.

## Abstract (web summary)

Senescent melanocytes of skin nevi drive hyperactivation of hair growth through the signalling factor SPP1.

## Main

Stem cells (SCs) are critically required for long-term tissue maintenance and regeneration. To perform their function, SCs remain quiescent and transiently activate only when warranted, a switch that is tightly controlled. Immediate control is exerted by the short-range signalling niche^1^. In addition, activities of thousands of individual SC niches are coordinated by long-range signalling cues from the surrounding tissues^5^. Because long-range signals coordinate activities of many SC niches at once, any changes in them can profoundly alter the overall regenerative potential of an organ. However, which cell types can function as efficient long-range regulators of SCs is poorly understood.

Skin offers a valuable model system for studying these fundamental aspects of SC biology. Skin contains progenitor-rich hair follicles (HFs) that renew in cycles^6^. Each cycle starts with SC activation^7^ and requires signalling by the niche, featuring specialized dermal papilla fibroblasts^8^. Although in principle HFs are able to renew cyclically without external signalling inputs, many thousands of HFs physiologically coordinate their hair-making activities for the common goal of proper fur ‘manufacturing’^9^. Coordination is achieved via shared signalling between neighbouring HFs^10^ and other non-hair skin cell types. The most prominent effects on hair renewal are exerted by skin adipocytes^11^ and adipose progenitors^12^. This is possible because HFs and adipose tissue are close to each other and because they use some of the same signalling pathways—WNT, BMP, Hedgehog and PDGF—to regulate their cellular lineages. Innate and adaptive immune cells are also potent modifiers of hair growth dynamics^13,14^.

**Fig. 1:**
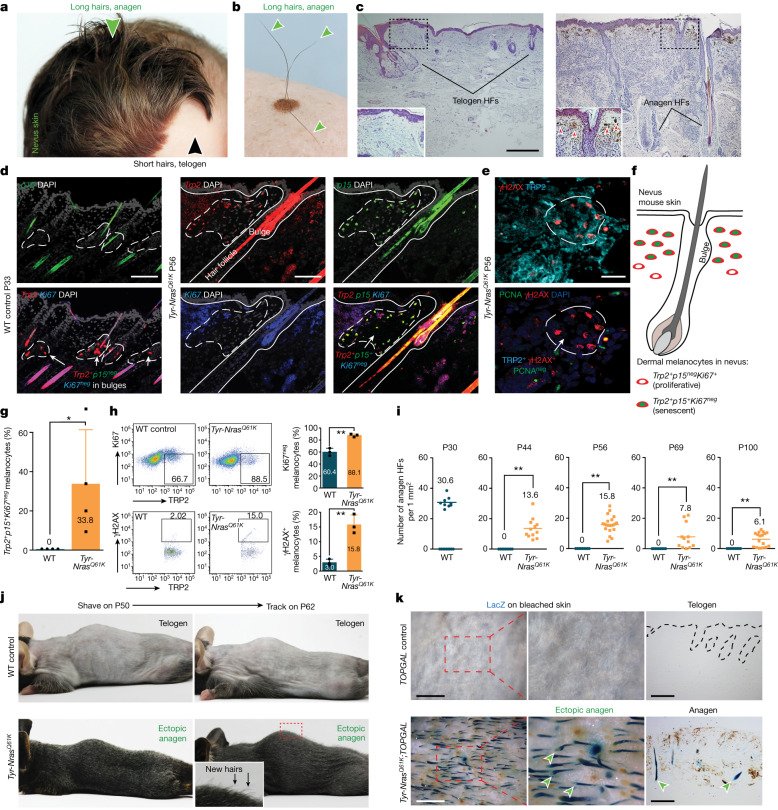
Hyperactivation of hair growth in nevus skin. **a**,**b**, Hair growth (arrowheads) is enhanced within congenital (7-month old; **a**) and acquired (42-year old; **b**) melanocytic nevi in humans. **c**, Facial HFs that commonly remain in telogen in normal skin (left) activate and enter new anagen in nevus skin (right). The red arrowheads mark dermal melanin. **d**,**f**,**g**, Compared with P33 WT anagen skin, P56 *Tyr-Nras*^*Q61K*^ skin contained clusters of *Trp2*^*+*^*p15*^*+*^*Ki67*^*neg*^ melanocytes in the upper dermis. In **g,** *n* = 4; *P* = 0.0455668. **e**, P56 *Tyr-Nras*^*Q61K*^ skin contained clusters of TRP2^+^γH2AX^+^PCNA^neg^ dermal melanocytes. **h**, Compared with P30 WT anagen skin, P56 *Tyr-Nras*^*Q61K*^ skin showed significantly increased numbers of TRP2^+^Ki67^neg^ (*n* = 3; *P* = 0.0019135) and TRP2^+^γH2AX^+^ melanocytes on cytometry (*n* = 3; *P* = 0.0028236). **i**–**k**, *Tyr-Nras*^*Q61K*^ mice displayed enhanced hair growth. At all postnatal time points examined (also see Extended Data Fig. 1), *Tyr-Nras*^*Q61K*^ skin contained many ectopic anagen HFs. Anagen HFs are quantified (**i**). In **i**, *n* = 9 at P30, *n* = 12 (*P* = 0.0000108) at P44, *n* = 21 (*P* = 0.0000000000183) at P56, *n* = 12 (*P* = 0.00329) at P69 and *n* = 17 (*P* = 0.0000239) at P100. In **j**, 12 days after shaving at P50, many new hairs grew in *Tyr-Nras*^*Q61K*^, but not in WT mice. In **k**, at P56, *Tyr-Nras*^*Q61K*^;*TOPGAL* mice, but not control *TOPGAL* mice, showed many lacZ^+^ anagen HFs (arrowheads). In **g**–**i**, *n* refers to biologically independent samples. Data are mean ± s.d. *P* values were calculated using unpaired one-tailed (**g**,**i**) or two-tailed (**h**) Student’s *t*-test. *P ≤ 0.05 and **P ≤ 0.01. Scale bars, 20 μm (**e**), 100 μm (**d**), 500 μm (**c**), 1 mm (wholemount; **k**) and 200 μm (histology; **k**). The image in part **a** is reproduced with permission from S. Liber. Source data

Because cyclic hair renewal is tightly controlled at the level of SC quiescence, naturally occurring conditions of excessive hair growth are rare. Hairy pigmented nevi, both congenital (Fig. 1a,c) and acquired (Fig. 1b), are a type of benign skin lesion in humans that can show prominent hair growth. Despite being well known clinically, the mechanism behind excessive hair growth in nevi is not understood. Oncogene mutations, commonly in *Nras* (also known as *Alps4*) or *Braf*, in skin melanocytes induce nevi^15^. Mutant cells first transiently expand but subsequently activate oncogene-induced senescence (OIS)^16^, giving rise to a spatially restricted lesion enriched for senescent cells. Once in full senescence, cells express a specialized secretome: the senescence-associated secretory phenotype (SASP)^17^. Several inflammatory cytokines and growth factors are part of the SASP, and their essential signalling roles are being rapidly recognized in normal embryonic development^18^, cellular reprogramming^19^, injury repair^20^ and cancer progression^21,22^. We hypothesized that enhanced hair growth in hairy nevi is driven by activating signalling from dermal clusters of senescent melanocytes to HF SCs.

## Senescent cells activate hair growth

**Extended Data Fig. 1:**
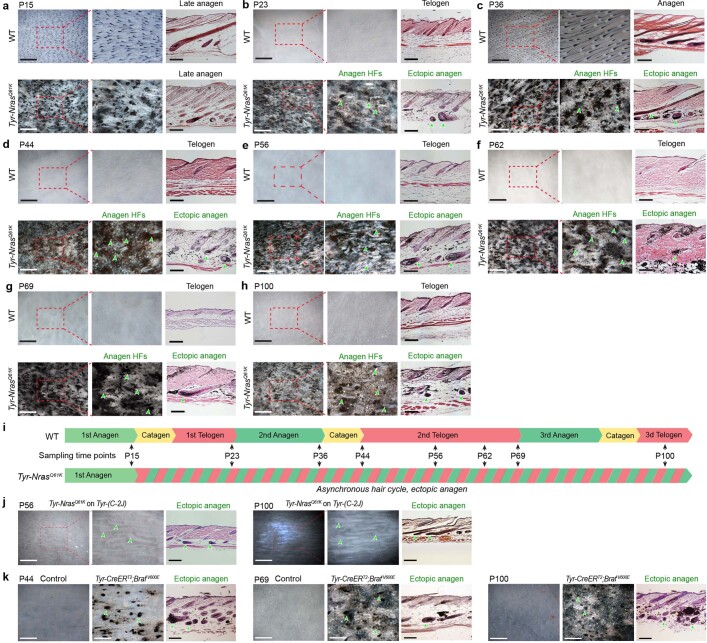
Nevus mouse models exhibit ectopic hair growth. **a–h**, At all postnatal time points examined, *Tyr-Nras*^*Q61K*^ mice showed ectopic anagen HFs. In WT control mice, HFs are in first anagen at day P15 (**a**); in first telogen at P23 (**b**); in second anagen at P36 (**c**); in extended second telogen at P44 (**d**), P56 (**e**), P62 (**f**), P69 (**g**); and in third telogen at P100 (**h**). In contrast, at all of the above time points, *Tyr-Nras*^*Q61K*^ skin contained many ectopic anagen HFs (green arrowheads). For each time point, representative wholemount (left) and histology samples (right) are shown. **i**, Schematic representation of the hair cycle state in WT control mice (top) and *Tyr-Nras*^*Q61K*^ mice (bottom) at indicated time points (middle). Colors: green – anagen, yellow – catagen, red – telogen. **j**, Albino *Tyr-Nras*^*Q61K*^ mice (crossed onto an albino *Tyr(C-2J)* background) maintain ectopic hair growth phenotype (green arrowheads) both at P56 (left) and P100 (right). For each time point, representative wholemount and histology samples are shown. **k**, *Tyr-CreER*^*T2*^*;Braf*^*V600E*^ mice induced with tamoxifen at P2-4 formed nevi and exhibited ectopic hair growth. At all postnatal time points examined (P44, P69 and P100), dorsal skin in induced *Tyr-CreER*^*T2*^*;Braf*^*V600E*^ mice contained many ectopic anagen HFs (green arrowheads). In contrast, HFs in induced control mice at the above time points were in telogen. For each time point, representative wholemount and histology samples (for *Tyr-CreER*^*T2*^*;Braf*^*V600E*^ mice) are shown. Also, see Extended Data Fig. 4a,b. Scale bars, **a–h, j** (wholemount) – 1 mm; **k** (wholemount) – 500; **a–h, j, k** (histology) – 200 μm.

First, we asked whether mouse models for melanocytic nevi replicate enhanced hair growth. We studied two established models: constitutive *Tyr-Nras*^*Q61K*^ mice^2^, which model congenital nevi, and inducible *Tyr-CreER*^*T2*^;*Braf*^*V600E*^ mice^3^, which model acquired nevi. In both models, oncogenes are overexpressed from the *Tyr* enhancer–promoter regulatory region that is highly specific to neural crest-derived melanocytes. Normal hair growth in mice is coordinated: large groups of HFs jointly transition from the resting phase (telogen) to the active growth phase (anagen) and then via the regression phase (catagen) back into telogen^9,11^. This coordination causes HF SCs to spend a large portion of their lifecycle in quiescence, only transiently activated to regenerate new hairs within discrete HF groups. Resting HFs house melanocyte SCs, located in the shared niche with epithelial SCs, whereas growing HFs also contain activated, pigment-producing melanocytes at their base. *Tyr-Nras*^*Q61K*^ mice, whose dermis but not HFs themselves become populated by senescent melanocytes identified as non-proliferative *p15*^*+*^ (Fig. 1d,f,g) and non-proliferative γH2AX^+^ melanocytes (Fig. 1e,h), showed dramatically accelerated hair growth, with many ectopic anagen HFs present at any given time (*n* = 3 per time point) (Fig. 1i–k and Extended Data Fig. 1). In control mice, dorsal HFs were in first anagen at postnatal day 15 (P15) (Extended Data Fig. 1a), first telogen by P23 (Extended Data Fig. 1b) and second anagen by P36 (Extended Data Fig. 1c). After that, HFs entered a lengthy second telogen spanning P44–P69 (Extended Data Fig. 1d–g). By contrast, at all time points examined, *Tyr-Nras*^*Q61K*^ skin contained ectopic anagen HFs (Fig. 1i,j and Extended Data Fig. 1), which were numerous even at P100 (Extended Data Fig. 1h). The ectopic anagen phenotype was especially visible in *Tyr-Nras*^*Q61K*^;*TOPGAL* mice (*n* = 4), where all anagen HFs strongly activated the *TOPGAL* WNT reporter and stained positive for lacZ (Fig. 1k). Ectopic anagen HF density in *Tyr-Nras*^*Q61K*^ mice varied between the time points, but on average it was 35.4% relative to synchronous anagen HF density in P30 wild-type (WT) skin (Fig. 1i). We crossed *Tyr-Nras*^*Q61K*^ mice onto an albino *Tyr*(*C-2J*) background carrying a mutation in the *Tyr* gene. Despite the lack of melanin, albino *Tyr-Nras*^*Q61K*^ mice displayed ectopic anagen at both P56 and P100 (Extended Data Fig. 1j), indicating that it is not excessive melanogenesis but rather senescent melanocytes that are necessary for the nevus hair phenotype.

**Extended Data Fig. 2:**
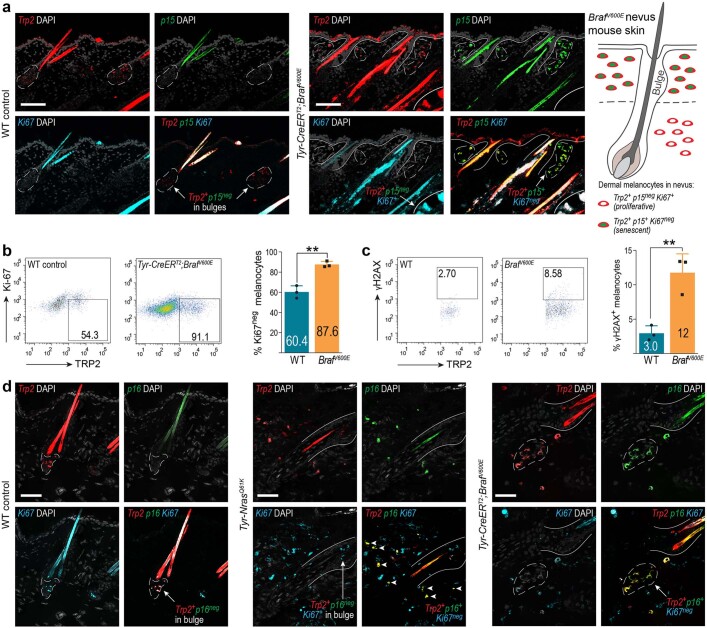
Nevus melanocytes but not normal melanocytes are sufficient to activate hair growth. **a**, Compared to skin from control mice, skin from P69 *Tyr-CreER*^*T2*^*;Braf*^*V600E*^ mice induced with tamoxifen at P21-25, contained clusters of *Trp2*^*+*^*/p15*^*+*^*/Ki67*^*neg*^ melanocytes in upper dermis next to bulge regions of HFs. **b, c**, Compared to skin from control mice, skin from P69 *Tyr-CreER*^*T2*^*;Braf*^*V600E*^ mice induced at P21-25, contained significantly more TRP2^+^/Ki67^neg^ melanocytes (**b**) and TRP2^+^/γH2AX^+^ melanocytes (**c**). Left – representative cytometry plots, right – quantification. In **b**, *n* = 3; *P* = 0.00239. In **c**, *n* = 3; *P* = 0.00675. **d**, Compared to skin from control mice (left), skin from *Tyr-Nras*^*Q61K*^ mice (center) and *Tyr-CreER*^*T2*^*;Braf*^*V600E*^ mice (right) contained clusters of *Trp2*^*+*^*/p16*^*+*^*/Ki67*^*neg*^ melanocytes in upper dermis next to bulge regions of HFs. In **b, c**, *n* = independent experiments. *P* values are calculated using unpaired two-tailed Student’s t-test. **P ≤ 0.01. Scale bars, **d** – 50 μm; **a** – 100 μm. Source data

**Extended Data Fig. 3:**
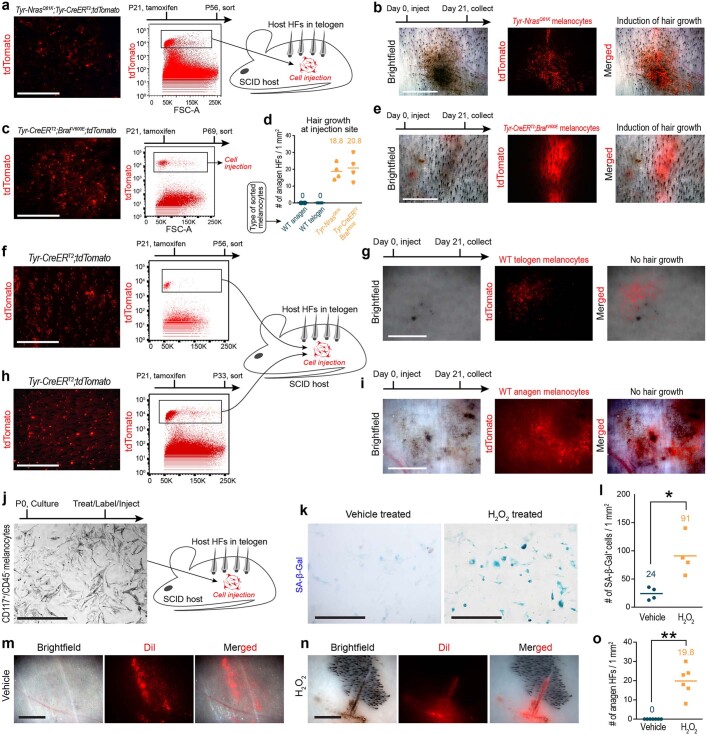
Nevus melanocytes and senescent melanocytes stimulate new hair growth. **a–i**, Melanocyte grafting experiments, in which melanocyte lineage cells were isolated as tdTomato^+^ cells from mice that contain *Tyr-CreER*^*T2*^ and *tdTomato* constructs, and that were induced with tamoxifen at P21. **a–e**, tdTomato^+^ melanocytes were isolated from P56 *Tyr-Nras*^*Q61K*^*;Tyr-CreER*^*T2*^*;tdTomato* (**a**) and P69 *Tyr-CreER*^*T2*^*;Braf*^*V600E*^*;tdTomato* nevus mouse skin (**c**) and injected into *SCID* mouse skin. Both types of mutant melanocytes (**b, e**) induced anagen after 21 days. **f–i**, Control melanocyte lineage cells were isolated from *Tyr-CreER*^*T2*^*;tdTomato* mice during telogen at P56 (**f**) and during anagen at P33 (**h**) and intradermally injected into telogen skin of *SCID* mice. Cells from both conditions (**g, i**) did not induce ectopic anagen 21 days after injection. Representative samples are shown in (**b, e, g, i**). Anagen HFs for experiments from (**b, e, g, i**) are quantified in (**d**). In **d**, *n* = 4. **j–o**, H_2_O_2_-induced senescence experiment (**j**). Senescent status of H_2_O_2_-treated melanocytes was confirmed with senescent β-Gal staining (**k, l**). H_2_O_2_-treated (**n**), but not control DiI-labeled melanocytes (**m**) induced anagen 21 days after injection into *SCID* mice. Anagen HFs are quantified in (**o**). In **l**, *n* = 4; *P* = 0.0112. In **o**, *n* = 7 in vehicle, *n* = 6 in H_2_O_2_; *P* = 0.000024. In **d, l, o**, *n* = biologically independent samples. *P* values are calculated using unpaired two-tailed Student’s t-test. *P ≤ 0.05, **P ≤ 0.01. Scale bars, **m, n** – 1 mm; **a, b, c, e, f–i** – 2 mm; **j, k** – 200 μm. Source data

**Extended Data Fig. 4:**
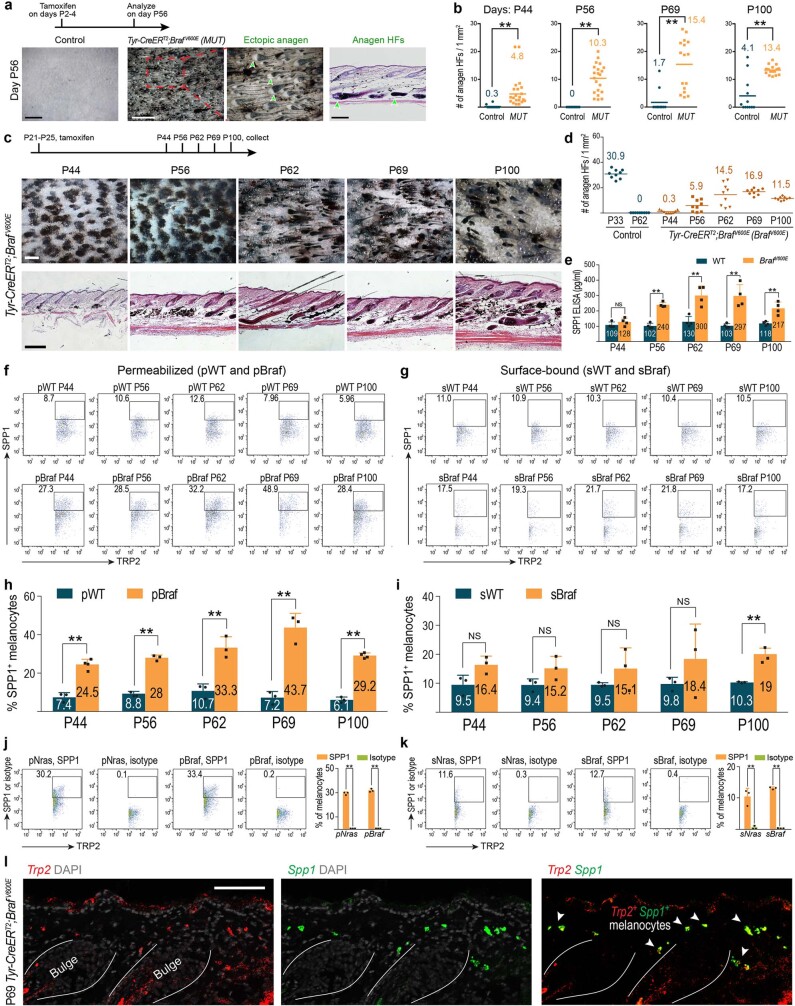
Induction of nevi results in hair growth hyper-activation and osteopontin overexpression. **a-b**, Compared to control, *Tyr-CreER*^*T2*^*;Braf*^*V600E*^ mice induced with tamoxifen at P2-4, showed prominent hair growth. Representative P56 skin samples are shown in (**a**). Anagen HFs are marked in **(a)** and quantified in (**b**). In **b**, at P44 (*n* = 12 in control, *n* = 20 in mutant; *P* = 0.00218), at P56 (*n* = 12 in control, *n* = 21 in mutant; *P* = 0.00000000804), at P69 (*n* = 12 in control, *n* = 16 in mutant; *P* = 0.0000526), at P100 (*n* = 12 in control, *n* = 16 in mutant; *P* = 0.00000662). **c, d**, Following tamoxifen-induction at P21-25, *Tyr-CreER*^*T2*^*;Braf*^*V600E*^ mice developed nevi by P44 and started to display ectopic hair growth from P56 onward. Representative wholemount and histology samples at five time points between P44-100 are shown in (**c**), and anagen HF density quantification is shown in (**d**). In **d**, *n* = 9. **e**, On ELISA, SPP1 levels became significantly higher in the supernatant from day 5 cultures of primary sorted *Tyr-CreER*^*T2*^*;Braf*^*V600E*^ melanocytes at five indicated timepoints from P56 onward. Data from P69 cells is also shown in main Fig. 3e. In **e**, at P44 (*n* = 4; *P* = 0.2686), at P56 (*n* = 4; *P* = 0.0000269), at P62 (*n* = 4; *P* = 0.003095), at P69 (*n* = 4; *P* = 0.00224), at P100 (*n* = 4; *P* = 0.0035). **f-i**, On cytometry, SPP1 levels in permeabilized cells (**f, h**) as well as surface-bound SPP1 levels in non-permeabilized cells (**g, i**) were significantly higher in melanocytes from induced *Tyr-CreER*^*T2*^*;Braf*^*V600E*^ mice (pBraf, sBraf) compared to wild type control mice (pWT, sWT) at indicated time points. Representative cytometry plots are shown in (**f, g**) and quantification is show in (**h, i**). Data from P69 cells is also shown in main Fig. 3d. In **h**, at P44 (*n* = 3 for pWT, *n* = 4 for pBraf; *P* = 0.000318), at P56 (*n* = 3; *P* = 0.0000533), at P62 (*n* = 3; *P* = 0.00426), at P69 (*n* = 3; *P* = 0.001397), at P100 (*n* = 3 for pWT, *n* = 4 for pBraf; *P* = 0.00000386). In **i**, at P44 (*n* = 3; *P* = 0.0531), at P56 (*n* = 3; *P* = 0.0912), at P62 (*n* = 3; *P* = 0.2495), at P69 (*n* = 3; *P* = 0.291), at P100 (*n* = 3; *P* = 0.00399). **j–k**, Cytometry of permeabilized (**j**) and non-permeabilized melanocytes (**k**) showed significantly higher levels of SPP1 compared to isotype control both in *Tyr-Nras*^*Q61K*^ mice (pNras, sNras) and *Tyr-CreER*^*T2*^*;Braf*^*V600E*^ mice (pBraf, sBraf). Representative cytometry plots are shown on the left and quantification on the right of **j** and **k**. In **j**, for pNras (*n* = 3; *P* = 0.00000175), for pBraf (*n* = 3; *P* = 0.00000213). In **k**, for sNras (*n* = 3; *P* = 0.00297), for sBraf (*n* = 3; *P* = 0.000000536). **l**, Skin of P69 *Tyr-CreER*^*T2*^*;Braf*^*V600E*^ mice contained *Trp2*^*+*^*/Spp1*^*+*^ melanocytes in upper dermis adjacent to bulge regions of HFs. In **b, d**, *n* = biologically independent samples. In **e, h, i, j, k**, *n* = independent experiments. Data are mean ± s.d. *P* values are calculated using unpaired one-tailed (in **b** at P56) or two-tailed (in **b** at P44, P69, P100, **e, h, i, j, k**) Student’s t-test. NS, *P* ≥ 0.05, *P ≤ 0.05, **P ≤ 0.01. Scale bars, **a** (wholemount) – 1 mm; **c** (wholemount) – 300 μm; **c** (histology) – 200 μm; **a** (histology), **l** – 100 μm. Source data

**Extended Data Fig. 5:**
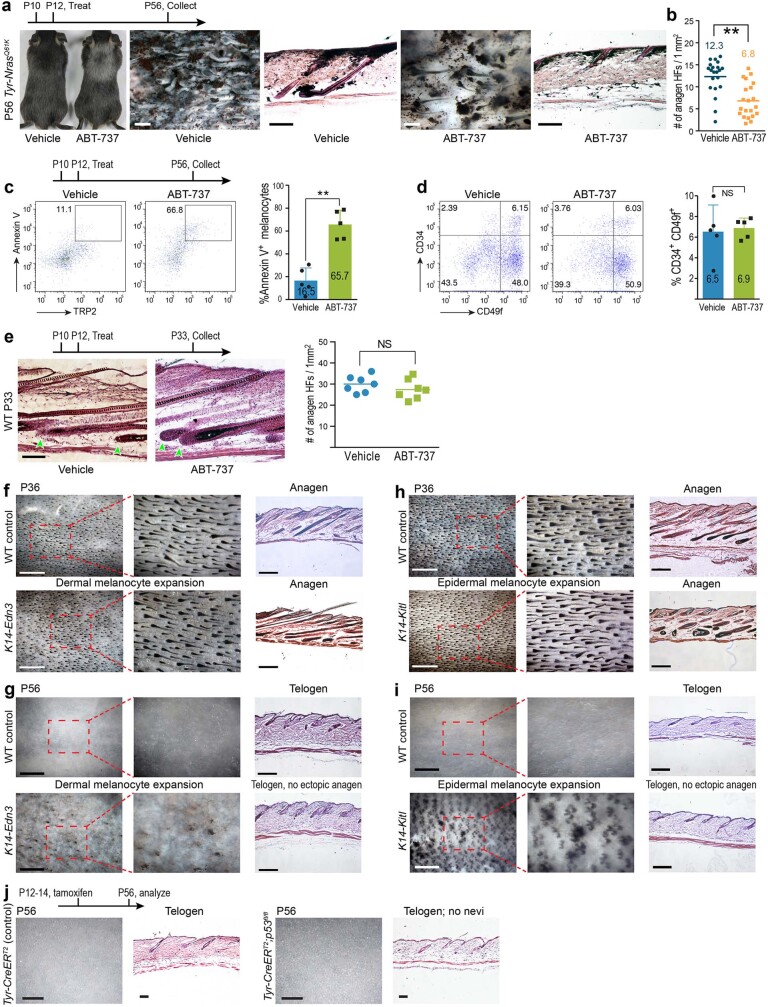
Effect of ABT-737 treatment and non-nevus melanocyte expansion on hair cycle. **a-b**, Unlike vehicle, subcutaneous ABT-737 treatment of *Tyr-Nras*^*Q61K*^ mice at P10 and P12 decreased fur pigmentation and reduced anagen HFs at P56 (**a**). Anagen HFs are quantified in (**b**). In **b**, *n*=21; *P* = 0.0000454. **c-e**, Effect of ABT-737 treatment on melanocytes, bulge stem cells and hair cycle status. **c**, On cytometry at P56, the percentage of TRP2^+^/Annexin V^+^ melanocytes in *Tyr-Nras*^*Q61K*^ mice significantly increased in response to ABT-737 treatment at P10-12. In **c**, *n* = 5; *P* = 0.0001816. **d**, On cytometry at P56, the abundance of CD34^+^/CD49f^+^ bulge stem cells in *Tyr-Nras*^*Q61K*^ mice was unchanged by ABT-737 treatment at P10-12. In **d**, *n* = 5; *P* = 0.7891838. **e**, ABT-737 treatment at P10-12 did not affect normal anagen timing in WT mice – skin from both vehicle and ABT-737 treated animals contained HFs in anagen at P33. In **e**, *n* = 7; *P* = 0.2898739. In (**c, d, e**) representative data is shown on the left, and data quantification – on the right. **f–j**, Mice with non-nevus expansion in melanocytes display normal hair cycle timing. **f–i**, Similar to control mice, *K14-Edn3* mice with dermal melanocyte expansion (**f, g**) and *K14-Kitl* mice with epidermal melanocyte expansion (**h, i**) were in synchronized anagen at P36 (**f, h**) and synchronized telogen at P56 (**g, i**). **j**, After tamoxifen induction at P12-14, *Tyr-CreER;p53*^*fl/fl*^ mice with melanocyte-specific deletion of *p53*, did not form nevi and exhibited telogen HFs at P56, analogous to induced control mice. In **b**, *n* = biologically independent samples. In **c, d, e** *n* = independent experiments. Data are mean ± s.d. *P* values are calculated using unpaired two-tailed Student’s t-test. NS, *P* ≥ 0.05, *P ≤ 0.05, **P ≤ 0.01. Scale bars, **f–j** (wholemount) – 1 mm; **a, f–j** (histology) – 200 μm; **e** – 100 μm. Source data

Next, we modelled early acquired nevi in *Tyr-CreER*^*T2*^;*Braf*^*V600E*^ mice that were treated with tamoxifen either early at P2–P4 or late at P21–P25. Unlike induced control animals, induced mutant mice accumulated clusters of senescent non-proliferative *p15*^*+*^ (Extended Data Fig. 2a), non-proliferative γH2AX^+^ (Extended Data Fig. 2b,c) and non-proliferative *p16*^*+*^ (Extended Data Fig. 2d) melanocytes in the dermis adjacent to HFs. Mutant mice induced at P2–P4 displayed prominent ectopic anagen at P44, P56, P69 and P100 (*n* = 4 per time point) (Extended Data Figs. 1k and 4a,b). Across time points, they averaged 35.7% anagen HFs relative to P30 WT skin, which closely phenocopied congenital *Tyr-Nras*^*Q61K*^ mutants. Likewise, mutant mice treated with tamoxifen at P21–P25 also showed prominent ectopic anagen starting at P56 (*n* ≥ 3 per time point) (Extended Data Fig. 4c,d). We also asked whether injection of nevus-derived melanocytes into normal telogen skin would be sufficient to induce ectopic anagen. We sorted tdTomato^+^ melanocyte lineage cells from the skin of congenital *Tyr-Nras*^*Q61K*^;*Tyr-CreER*^*T2*^;*tdTomato* (Extended Data Fig. 3a) and acquired *Tyr-CreER*^*T2*^;*Braf*^*V600E*^;*tdTomato* mice (Extended Data Fig. 3c). Intradermal injection of sorted cells from both nevus mouse models into telogen skin of *SCID* mice (*n* = 4 each) induced new anagen within 21 days (Extended Data Fig. 3b,e), albeit their continued senescent status at the grafted site was not verified. Yet, by contrast, injection of sorted cells from control *Tyr-CreER*^*T2*^;*tdTomato* mice isolated during both telogen (P56) and anagen (P33) did not activate new anagen in *SCID* host skin (*n* = 4 each) (Extended Data Fig. 3d,f–i). We also generated senescent β-galactosidase-positive (β-Gal^+^) melanocytes by exposing primary CD117^+^ newborn mouse melanocytes to H_2_O_2_ in vitro (Extended Data Fig. 3j–l). Unlike control cultured melanocytes (*n* = 7), DiI-labelled H_2_O_2_-treated melanocytes induced new anagen in telogen *SCID* skin 21 days after injection (*n* = 6) (Extended Data Fig. 3m–o). We also subcutaneously treated mice with the small-molecule BCL-2 inhibitor ABT-737, which in P56 *Tyr-Nras*^*Q61K*^ mice, induced prominent apoptosis of melanocytes but did not affect the abundance of HF SCs (*n* = 5) (Extended Data Fig. 5c,d), and in P33 WT mice did not delay normal anagen timing (*n* = 7) (Extended Data Fig. 5e). By contrast, ABT-737 treatment of *Tyr-Nras*^*Q61K*^ mice significantly reduced ectopic anagen HFs at P56 (*n* = 6) (Extended Data Fig. 5a,b), which we attribute to nevus melanocyte depletion. Next, we studied *K14-Edn3* and *K14-Kitl* mice, which, respectively, showed expansion in dermal and epidermal melanocytes that is not driven by oncogene mutation. Both mouse models showed normal hair cycle progression, with synchronized anagen at P36 (*n* = 3 per model) and synchronized telogen at P56 (*n* = 3 per model) (Extended Data Fig. 5f–i). Last, we induced *Trp53* (also known as *p53*) deletion in melanocytes, which despite being an oncogenic stimulation, did not induce OIS^17^, unlike *Nras*^*Q61K*^ or *Braf*^*V600E*^ overexpression. Analogous to control mice, HFs in tamoxifen-treated P56 *Tyr-CreER*^*T2*^;*Trp53*^*fl/fl*^ mice remained in telogen (*n* = 3) (Extended Data Fig. 5j). Together, our data show that congenital and acquired mouse models for melanocyte OIS reproduce the enhanced hair growth that is clinically observed in human hairy pigmented nevi and that senescent dermal melanocytes, but not normal melanocytes, are necessary and sufficient to hyperactivate HF renewal.

## Senescence disrupts SC quiescence

**Fig. 2:**
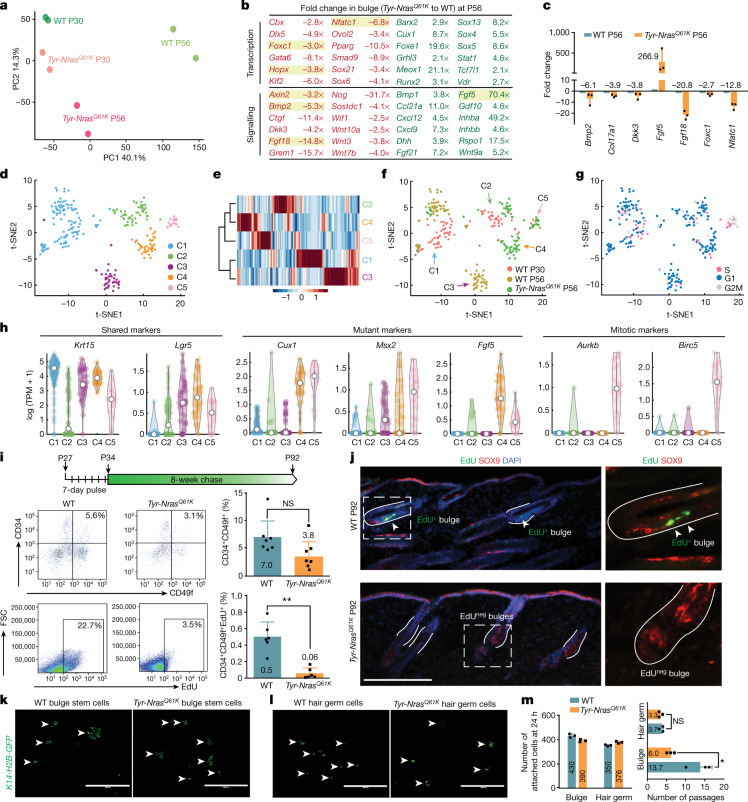
Hair SCs within nevus skin lose quiescence. **a**, On RNA-seq analysis, *Tyr-Nras*^*Q61K*^ bulge SCs differ from P30 and P56 WT bulge SCs. A principal component analysis plot is shown. See Extended Data Fig. 6. **b**, A list of selected downregulated (red) and upregulated (green) genes at P56 and *Tyr-Nras*^*Q61K*^ to WT fold change values. **c**, qRT–PCR of selected differentially expressed genes from **a**. *n* = 3. **d**, t-Distributed stochastic neighbour embedding (t-SNE) analysis on single-cell RNA-seq data for P30 and P56 WT and P56 *Tyr-Nras*^*Q61K*^ bulge SCs. Cells form five clusters: C1 to C5. **e**, Cladogram showing relative cluster similarity. **f**, t-SNE plot colour-coded by sample source. **g**, t-SNE plot colour-coded by inferred cell cycle state. **h**, Violin plots for selected genes. See Extended Data Fig. 6. TPM, transcripts per million. **i**, EdU pulse-chase analysis on bulge SCs. Unlike total numbers of CD34^+^CD49f^+^ bulge SCs (top), their EdU^+^ label-retaining subset reduced significantly in *Tyr-Nras*^*Q61K*^ versus control mice (bottom). *n* = 7 (*P* = 0.061857) for CD34^+^CD49f^+^ SCs and *n* = 6 (*P* = 0.0002048) for CD34^+^CD49f^+^EdU^+^ SCs. See Extended Data Fig. 7. FSC, forward scatter. **j**, Unlike WT, *Tyr-Nras*^*Q61K*^ HFs from part **i** lacked EdU^+^SOX9^+^ bulge SCs (yellow). **k**,**l**, Attachment rates for the *K14-H2B-GFP*^*+*^ bulge (**k**) and hair germ (**l**) cells were compatible between WT and *Tyr-Nras*^*Q61K*^ mice. Arrowheads mark cell colonies. **m**, Compared with WT, *Tyr-Nras*^*Q61K*^ bulge SCs prominently reduced serial passaging potential, whereas it was unaltered for hair germ progenitor cells. *n* = 3 (*P* = 0.5185185) for hair germ cells and *n* = 3 (*P* = 0.0168963) for bulge cells. In **c**,**i**,**m**, *n* refers to independent experiments. *P* values were calculated using unpaired two-tailed Student’s *t*-test. Not significant (NS), *P* ≥ 0.05, *P ≤ 0.05 and **P ≤ 0.01. Scale bars, 100 μm (**j**) and 1 mm (**k**,**l**). Source data

**Extended Data Fig. 6:**
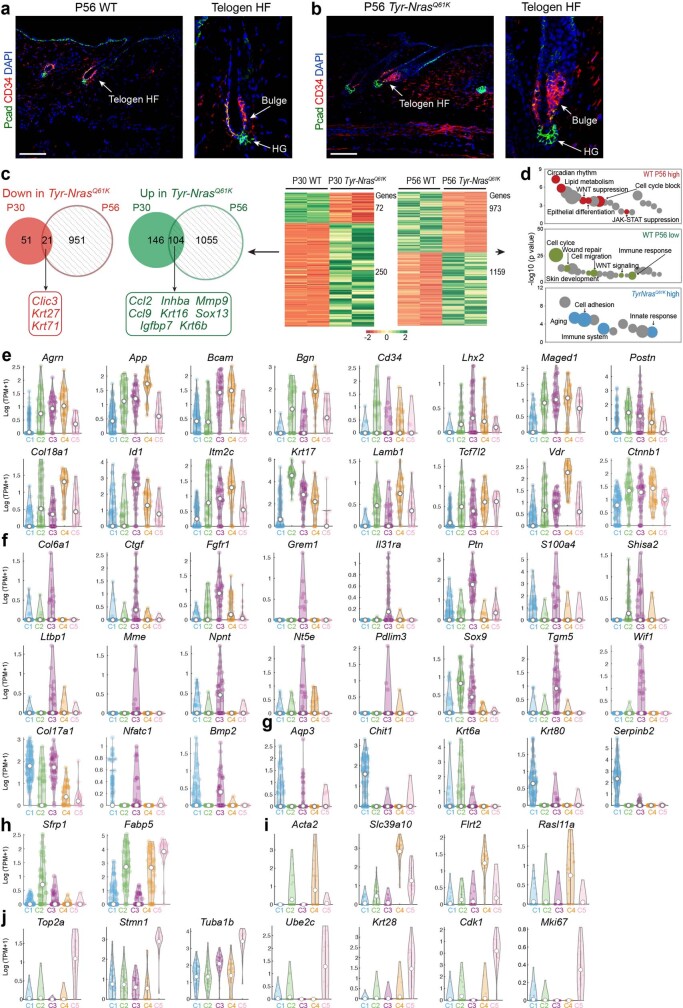
Gene expression patterns in *Tyr-Nras*^*Q61K*^ HFs stem cells. **a–b**, *Tyr-Nras*^*Q61K*^ telogen HFs maintain normal expression patterns of bulge and hair germ markers. Co-immunostaining for CD34 (red) and Pcad (green) showed that their expression pattern in *Tyr-Nras*^*Q61K*^ telogen HFs (**b**) is consistent with that in WT telogen HFs (**a**). Bulge cells are CD34^+^ and Pcad^low^, while hair germ (HG) cells are CD34^neg^ and Pcad^high^. **c–d**, RNA-seq analysis on WT and *Tyr-Nras*^*Q61K*^ bulge SCs. Venn diagrams and DEGs heatmap are shown in (**c**). Venn diagrams identify 21 downregulated and 104 upregulated genes specific to *Tyr-Nras*^*Q61K*^. Bubble charts in (**d**) show enriched (red) and depleted (green) GO terms in P56 WT bulge SCs, and enriched GO terms in *Tyr-Nras*^*Q61K*^ bulge SCs (blue). **e–j**, Gene expression patterns in bulge stem cells on single-cell RNA-seq. Cell clusters are color-coded according to main Fig. 2d and are as follows: C1 – inner bulge cells, present in P30 and P56 WT samples and in P56 *Tyr-Nras*^*Q61K*^ sample; C2 – anagen-specific outer bulge cells, present in P30 WT and P56 *Tyr-Nras*^*Q61K*^ samples; C3 – telogen-specific outer bulge cells, present in P56 WT sample; C4 and C5 – outer bulge cells specific to *Tyr-Nras*^*Q61*^ sample. Violin plots are shown with normalized expression values along the Y-axis. **e**, Expression patterns of outer bulge markers, showing enrichment in clusters C2, C3 and C4. **f**, Expression patterns of telogen-phase outer bulge markers, showing enrichment in cluster C3. **g**, Expression patterns of inner bulge markers, showing enrichment in cluster C1. **h**, Expression patterns of anagen-phase enriched markers. **i**, Expression patterns of mutant-enriched markers in cluster C4. **j**, Expression patterns of mutant-enriched markers in cluster C5. Scale bars, **a, b** – 100 μm.

We next asked how bona fide HF bulge SCs are affected by the nevus environment. We profiled their transcriptomes by RNA sequencing (RNA-seq) at P30 and P56, when WT HFs are in anagen and telogen, respectively. Bulge SCs were isolated as GFP^+^CD34^+^Pcad^low^ cells both from *K14-H2B-GFP* control mice and *Tyr-Nras*^*Q61K*^;*K14-H2B-GFP* mutant mice, in which CD34 and Pcad maintain WT expression patterns (Extended Data Fig. 6a,b). RNA-seq revealed prominent gene expression differences between *Tyr-Nras*^*Q61K*^ and control bulge SCs (Fig. 2a, Extended Data Fig. 6c and Supplementary Table 1). The largest differences were seen at P56, with mutant SCs downregulating and upregulating 973 and 1,159 genes, respectively. Depleted gene ontology categories for mutant SCs included cell cycle block, circadian rhythm, and WNT and JAK–STAT suppression, whereas enriched categories contained cell cycle, cell migration, WNT signalling and skin development (Extended Data Fig. 6d and Supplementary Table 1). These gene ontology signatures indicate that *Tyr-Nras*^*Q61K*^ bulge SCs lose quiescence. At the gene level, multiple quiescence markers, including *Axin2*, *Bmp2*, *Col17a1*, *Ctgf*, *Fgf18*, *Foxc1*, *Grem1*, *Nfatc1* and *Wif1*, were downregulated in P56 *Tyr-Nras*^*Q61K*^ SCs (Fig. 2b,c and Supplementary Table 1).

To confirm that the *Tyr-Nras*^*Q61K*^ bulk RNA-seq signature is not being simply dominated by near-normal activated SCs from ectopic anagen HFs, we compared P56 mutant with P30 anagen and P56 telogen WT bulge cells by single-cell RNA-seq. WT cells from P30 and P56 formed the shared cluster C1 and two phase-specific clusters: anagen-specific C2 and telogen-specific C3 (Fig. 2d–f). Upon marker analysis, C1 cells matched the signature of inner bulge cells, which includes *Chit1*, *Krt6a* and *Krt80*, whereas both C2 and C3 cells matched that of outer bulge bona fide SCs, which includes *Col18a1*, *Krt17*, *Lhx2*, *Tcf7l2* and *Vdr*^23^ (Fig. 2h, Extended Data Fig. 6e–j and Supplementary Tables 2 and 3). P56 mutant bulge cells dramatically altered their composition relative to WT cells; some cells contributed to the shared inner bulge cluster C1, others to the WT anagen-specific outer bulge cluster C2, whereas many cells formed two new mutant-specific clusters C4 and C5, which retained a core outer bulge signature (Fig. 2f and Extended Data Fig. 6e). No mutant cells contributed to the WT telogen-specific outer bulge cluster C3, which has a quiescent gene expression signature, including *Bmp2*, *Col17a1*, *Ctgf*, *Grem1*, *Nfatc1*, *Tgm5* and *Wif1* (Extended Data Fig. 6f). Loss of quiescence by mutant-specific outer bulge SCs was further evident from inferred cell cycle analysis: C5 cells were exclusively in S and G2/M phases (Fig. 2g) with prominently upregulated mitotic markers (Fig. 2h and Supplementary Table 3). Given that *Tyr-Nras*^*Q61K*^ skin contains a mixture of anagen and telogen HFs, the disappearance of WT telogen-specific C3 outer bulge cells supports the loss of quiescence by mutant telogen SCs. Outer bulge marker similarities between clusters C2 to C5 suggest that in the presence of nevus melanocytes, normally quiescent telogen SCs transition to a uniquely activated state.

**Extended Data Fig. 7:**
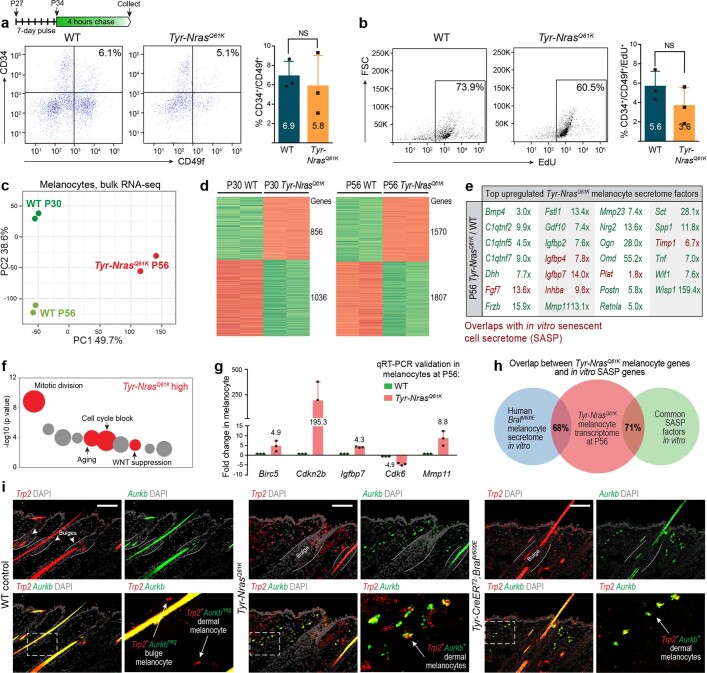
Characterization of bulge stem cells and melanocytes in nevus mouse models. **a, b**, Labeling efficiency of bulge stem cells after 7 days of EdU pulse was consistent between WT control and *Tyr-Nras*^*Q61K*^ mice. Representative cytometry plots (left) and data quantification (right) are shown for all CD34^+^/CD49f^+^ bulge stem cells in (**a**) and for their CD34^+^/CD49f^+^/EdU^+^ subset in (**b**). In **a**, *n* = 3; *P* = 0.63929124. In **b**, *n* = 3; *P* = 0.23549636. **c-f**, On RNA-seq, P56 *Tyr-Nras*^*Q61K*^ melanocytes differ from P30 and P56 WT melanocytes. PCA is shown in (**c**) and DEGs heatmap in **(d)**. Secretome factors upregulated in *Tyr-Nras*^*Q61K*^ melanocytes and *Tyr-Nras*^*Q61K*^/WT fold change values are shown in (**e**). Bubble chart showing enriched GO terms in *Tyr-Nras*^*Q61K*^ melanocytes are shown in (**f**). Selected bubbles are colored and annotated. **g**, qRT-PCR validation of selected differentially expressed genes from bulk RNA-seq data on melanocytes. In **g**, *n* = 3. **h**, Venn diagram showing the degree of overlap between the P56 *Tyr-Nras*^*Q61K*^ melanocyte transcriptome and published *in vitro* human senescent melanocyte secretome (blue, 68%) and core *in vitro* SASP factors (green, 71%). **i**, Compared to skin from wild type control mice (left), skin from *Tyr-Nras*^*Q61K*^ mice (middle) and induced *Tyr-CreER*^*T2*^*;Braf*^*V600E*^ mice (right) contained clusters of *Trp2*^*+*^*/Aurkb*^*+*^ melanocytes in upper dermis next to bulge regions of HFs. In **a, b**, *n* = independent experiments. Data are mean ± s.d. *P* values are calculated using unpaired two-tailed Student’s t-test. NS, *P* ≥ 0.05. Scale bars, **i** – 100 μm. Source data

Next, we confirmed loss of quiescence in functional assays. For pulse and pulse-chase experiments, which measure the cell cycle status of cells, mice were treated with EdU between P27 and P34, when WT HFs are in anagen and their SCs proliferate. Four hours after the EdU pulse, *Tyr-Nras*^*Q61K*^ mice displayed bulge SC labelling efficiency that was compatible with WT SCs (Extended Data Fig. 7a,b). However, in a pulse-chase assay, there was a prominent loss of EdU-retaining SCs in *Tyr-Nras*^*Q61K*^ mice as noted upon analysis at P92 (*n* = 4 per genotype) (Fig. 2i,j). We then performed a clonogenic assay, which measures long-term proliferative potential by cultured cells and identifies SCs on the basis of them being able to form large clones over many serial passages. We show that the attachment ability of *Tyr-Nras*^*Q61K*^ bulge SCs was similar to that of WT SCs, but their serial passaging potential was compromised; mutant SCs supported 6 passages (*n* = 3) compared with 13.7 passages for WT SCs (*n* = 3) (Fig. 2k,m). A decrease in passaging potential by bulge SCs indicates their faster proliferative exhaustion, a likely consequence of their long-term hyperproliferative status in vivo before culture. Attachment rates and passaging potential, however, did not differ between mutant (*n* = 3) and WT mice for hair germ cells, a short-lasting population of epithelial progenitors in telogen HFs (*n* = 3) (Fig. 2l,m).

## Osteopontin stimulates hair growth

Next, we asked which signalling factors are expressed by nevus melanocytes. We isolated the melanocyte lineage as tdTomato^+^ cells from the tamoxifen-induced *Tyr-Nras*^*Q61K*^;*Tyr-CreER*^*T2*^;*tdTomato* mutant and *Tyr-CreER*^*T2*^;*tdTomato* control skin. P56 mutant cells were compared with both P30 anagen and P56 telogen WT cells on bulk RNA-seq (Extended Data Fig. 7c,d). This strategy identified 598 mutant-specific upregulated genes, and also excluded genes regulated as part of the normal hair cycle. Mutant-specific genes were enriched for gene ontology terms, including ageing, WNT suppression, cell cycle block and mitotic division (Extended Data Fig. 7f and Supplementary Table 4). Consistent with dermal clusters of mutant melanocytes undergoing OIS, they upregulated tumour suppressors *Cdkn2b* (also known as *p15*), *Lzts1*, as well as *Cdkn3*, *H2afx* and the mitosis-associated genes *Aurka/b*, *Cdca3/8*, *Cdc20/25c*, *Cenpa*, *Mad2l1*, *Ncaph*, *Knstrn*, *Plk1*, *Psrc1* and *Reep4* (Extended Data Fig. 7g,i). Upregulation of mitosis-associated genes is consistent with the fact that oncogene-stimulated melanocytes enter OIS via a mitotic arrest pathway, rather that via G0 phase^24^. Focusing on the secretome, we identified 27 signalling factors specifically upregulated in nevus melanocytes, including the BMP members *Bmp4* and *Fstl1*, the WNT members *Frzb*, *Wif1* and *Wisp1*, the IGF regulators *Igfbp2/4/7*, as well as *Dhh*, *Fgf7*, *Spp1* (also known as osteopontin) and *Tnf* (Extended Data Fig. 7e). Of note, 68% of the secretome genes enriched in *BRAF*^*V600E*^-induced human senescent melanocytes in vitro^2^ and 71% of the core in vitro SASP factors^17^ were represented in the transcriptome of P56 *Tyr-Nras*^*Q61K*^ melanocytes (Extended Data Fig. 7h).

**Fig. 3:**
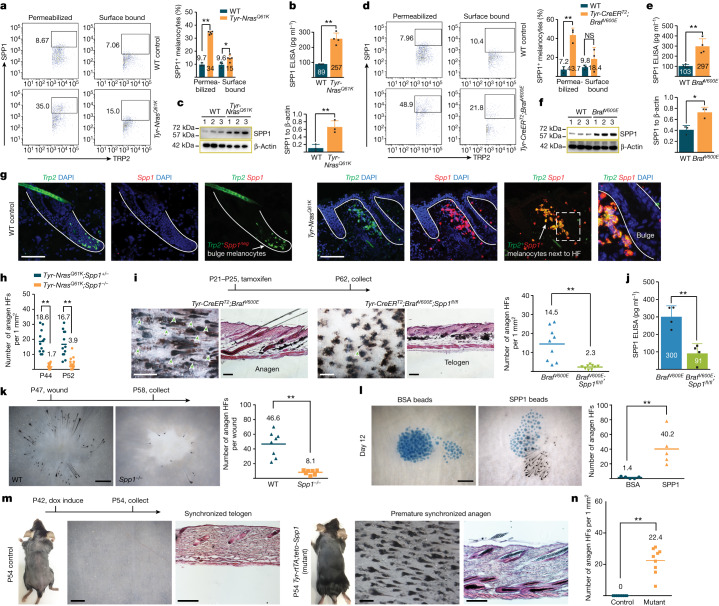
Secretome of nevus melanocytes contains SPP1 that promotes hair growth. **a**,**d**, On cytometry, SPP1 was increased in P56 *Tyr-Nras*^*Q61K*^ (**a**) and P69 *Tyr-CreER*^*T2*^;*Braf*^*V600E*^ (**d**) melanocytes. In **a**, for the permeabilized condition, *n* = 3 in WT and *n* = 5 in *Tyr-Nras*^*Q61K*^ (*P* = 0.000000115); for the surface-bound condition, *n* = 3 in WT and *n* = 5 in *Tyr-Nras*^*Q61K*^ (*P* = 0.0257). In **d**, for the permeabilized condition, *n* = 3 (*P* = 0.001397); for the surface-bound condition, *n* = 3 (*P* = 0.2888). See Extended Data Fig. 4. **c**,**f**, On western blot, SPP1 levels were increased in P56 *Tyr-Nras*^*Q61K*^ (**c**) and P69 *Tyr-CreER*^*T2*^;*Braf*^*V600E*^ (**f**) melanocytes. In **c**, *n* = 3; *P* = 0.00784. In **f**, *n* = 3; *P* = 0.0109. Uncropped gels are shown in Supplementary Fig. 1. **b**,**e**, On ELISA, SPP1 levels increased in day 5 cultures of P56 *Tyr-Nras*^*Q61K*^ (**b**) and P69 *Tyr-CreER*^*T2*^*;Braf*^*V600E*^ (**e**) melanocytes. In **b**, *n* = 3 in WT and *n* = 4 in *Tyr-Nras*^*Q61K*^; *P* = 0.00072. In **e**, *n* = 4; *P* = 0.00224. See Extended Data Fig. 4e. **g**, Unlike WT, *Tyr-Nras*^*Q61K*^ skin contained *Trp2*^*+*^*Spp1*^*+*^ melanocytes adjacent to HF bulges. **h**, Anagen HF quantification in *Tyr-Nras*^*Q61K*^;*Spp1*^−/−^ versus *Tyr-Nras*^*Q61K*^;*Spp1*^+/−^ control mice. At P44, *n* = 12 in control and *n* = 14 in *Tyr-Nras*^*Q61K*^;*Spp1*^−/−^ (*P* = 0.0000000191); at P56, *n* = 12 in control and *n* = 15 in *Tyr-Nras*^*Q61K*^;*Spp1*^−/−^ (*P* = 0.0000195). **i**, *Tyr-CreER*^*T2*^;*Braf*^*V600E*^;*Spp1*^*fl/fl*^ mice showed hair cycle quiescence rescue. Representative samples (left) and quantification (right) are displayed. *n* = 9; *P* = 0.000731. Arrowheads mark anagen HFs. **j**, On ELISA, SPP1 levels were reduced in day 5 cultures of *Tyr-CreER*^*T2*^;*Braf*^*V600E*^;*Spp1*^*fl/fl*^ versus *Tyr-CreER*^*T2*^;*Braf*^*V600E*^ melanocytes. *n* = 4; *P* = 0.00242. **k**, *Spp1*^−/−^ mice showed reduced wound-induced hair growth. Representative samples (left) and quantification (right) are displayed. *n* = 8 in WT and *n* = 7 in *Spp1*^−/−^; *P* = 0.0000575. **l**, Unlike BSA-soaked beads (blue), SPP1-soaked beads induced anagen in WT skin 12 days after injection. Representative samples (left) and quantification (right) are displayed. *n* = 5; *P* = 0.00562. **m**,**n**, Unlike control, doxycycline (dox)-treated P54 *Tyr-rtTA*;*tetO-Spp1* mice displayed premature anagen. Representative mice (**m**) and quantification (**n**) are displayed. In **n**, *n* = 9; *P* = 0.000000377. In **b**,**c**,**e**,**f**,**j**, *n* refers to independent experiments. In **a**,**d**,**h**,**i**,**k**,**l**,**n**, *n* refers to biologically independent samples. Data are mean ± s.d. *P* values were calculated using unpaired two-tailed Student’s *t*-test. NS, *P* ≥ 0.05, *P ≤ 0.05 and **P ≤ 0.01. Scale bars, 100 μm (**g**), 200 μm (histology; **i**,**m**) and 500 μm (wholemount; **i**,**k**,**l**,**m**). Source data

**Extended Data Fig. 8:**
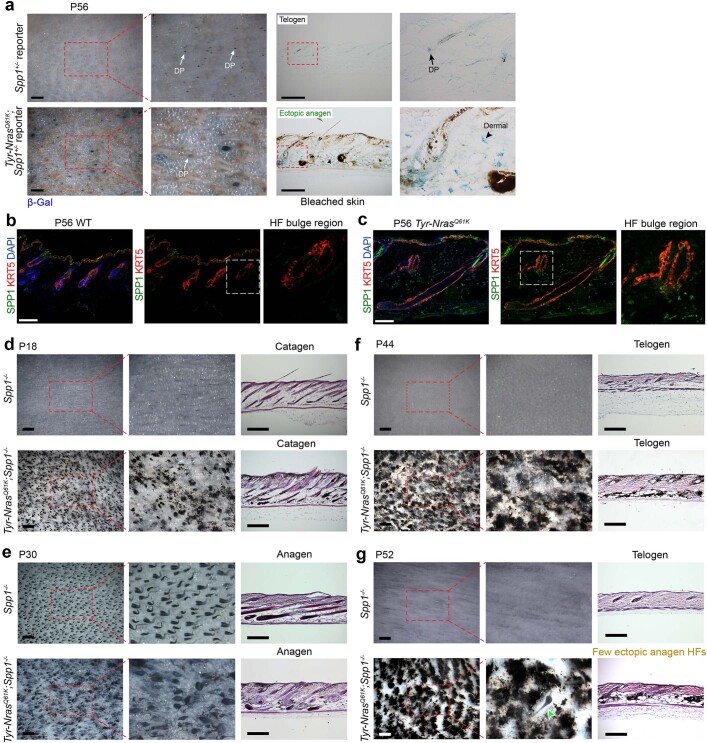
Changes in expression and the effect of osteopontin deletion in *Tyr-Nras*^*Q61K*^ skin. **a-c**, Osteopontin expression is increased in *Tyr-Nras*^*Q61K*^ skin. **a**, *Spp1* reporter activity was increased in *Tyr-Nras*^*Q61K*^ skin. LacZ staining (blue) on *Tyr-Nras*^*Q61K*^*;Spp1*^*+/−*^ *vs*. control *Spp1*^*+/−*^ P56 reporter mouse skin showed broad increase in LacZ^+^ cells. Dermal and dermal papilla (DP) expression sites are marked. For each panel, representative wholemount and histology samples are shown on the left and on the right, respectively. **b–c**, Co-immunostaining for KRT5 (red) and SPP1 (green) in P56 WT control (**b**) and *Tyr-Nras*^*Q61K*^ skin **(c)**. *Tyr-Nras*^*Q61K*^ skin showed prominently increased SPP1 expression in the dermal compartment, including around bulge regions of HFs (inserts). **d-g**, Osteopontin deletion rescues hair cycle quiescence in *Tyr-Nras*^*Q61K*^ mice. *Tyr-Nras*^*Q61K*^*;Spp1*^*−/−*^ mice showed rescue of hair cycle quiescence. Unlike *Tyr-Nras*^*Q61K*^ mice (see Extended Data Fig. 1), *Tyr-Nras*^*Q61K*^*;Spp1*^*−/−*^ mice showed synchronized catagen at P18 (**d**), synchronized anagen at P30 (**e**), synchronized telogen at P44 (**f**) and largely synchronized telogen P52 (**g**). For each time point, representative *Spp1*^*-/-*^ control and *Tyr-Nras*^*Q61K*^*;Spp1*^*-/-*^ mutant skin samples are shown. Wholemount samples are shown on the right and histology on the left of each panel. Scale bars, **b, c** – 100 μm; **a, d–g** (wholemount) – 500 μm; **a, d–g** (histology) – 200 μm.

*Spp1* was one of the topmost upregulated signalling transcripts in nevus melanocytes on RNA-seq. We confirmed this change at the protein level in sorted melanocytes from both the congenital and the acquired nevus mouse models. On cytometry, SPP1 levels were significantly increased in melanocytes from P56 *Tyr-Nras*^*Q61K*^ mice (*n* = 3) (Fig. 3a) and from tamoxifen-induced *Tyr-CreER*^*T2*^;*Braf*^*V600E*^ mice relative to control melanocytes at five time points between P44 and P100 (*n* = 3 each) (Fig. 3d and Extended Data Fig. 4f–i). Significantly increased SPP1 levels in P56 *Tyr-Nras*^*Q61K*^ and in P69 *Tyr-CreER*^*T2*^;*Braf*^*V600E*^ melanocytes were confirmed by western blot (*n* = 3 each) (Fig. 3c,f). Significant increase in SPP1 secretion was observed by ELISA on day 5 cultures of primary melanocytes sorted from P56 *Tyr-Nras*^*Q61K*^ mice (*n* = 3) (Fig. 3b) and from *Tyr-CreER*^*T2*^;*Braf*^*V600E*^ mice at four time points between P56 and P100 relative to control melanocyte cultures (*n* = 3 each) (Fig. 3e and Extended Data Fig. 4e). On staining, clusters of *Trp2*^*+*^*Spp1*^*+*^ melanocytes were observed in the upper dermis adjacent to bulge regions of HFs only in nevus mice, both congenital (Fig. 3g) and acquired (Extended Data Fig. 4l), but not in control mice. Consistent with published gene expression analyses^25^, lacZ staining in *Spp1*^+/−^ mice (which carry β-Gal knock-in) shows that *Spp1* expression in normal skin at homeostasis is very restricted, largely limited to dermal papilla fibroblasts of HFs (Extended Data Fig. 8a–c). Together, the above data support that SPP1 is an upregulated signalling factor in dermal clusters of nevus melanocytes.

Next, we asked whether SPP1 has a functional role in hairy nevus phenotype and whether it is sufficient to induce new hair growth. Using *Tyr-Nras*^*Q61K*^;*Spp1*^−/−^ mice, we showed that a germline loss-of-function mutation in *Spp1* is sufficient to rescue hair cycle quiescence in congenital nevus skin. Compared with *Tyr-Nras*^*Q61K*^ mice, whose HFs start cycling ectopically already at P23 (Extended Data Fig. 1b), ectopic anagen in *Tyr-Nras*^*Q61K*^;*Spp1*^−^^/−^ mice is largely prevented (*n* = 6 per time point) (Fig. 3h and Extended Data Fig. 8d–g). We also generated *Tyr-CreER*^*T2*^;*Braf*^*V600E*^;*Spp1*^*fl/fl*^ mice, in which tamoxifen treatment induces a conditional *Spp1* loss-of-function mutation in melanocytes along with oncogenic BRAF stimulation. We showed that melanocyte-specific *Spp1* deletion largely prevented ectopic hair cycle in P62 *Spp1*-deficient nevus mice compared with *Spp1*-intact nevus control animals (*n* = 5 each) (Fig. 3i), and that this correlated with a significant, approximately 70%, decrease in SPP1 secretion in primary melanocyte culture by ELISA (*n* = 3) (Fig. 3j). Partial SPP1 loss is attributed to incomplete efficiency of *CreER*-based recombination.

Unlike at homeostasis, SPP1 becomes prominently upregulated in skin wounds, both in wound fibroblasts^26^ and wound macrophages^27^. Considering this, we asked whether it mediates wound-induced hair growth phenomenon, when HFs at the wound margin enter premature anagen. Indeed, compared with WT mice (*n* = 8), *Spp1*^−/−^ mice showed significantly fewer anagen HFs at the margin of 5-mm wounds 11 days post-wounding (*n* = 7) (Fig. 3k). Ectopic anagen was prominently induced 12 days after intradermal injection of SPP1-soaked beads in WT mice compared with BSA-soaked control beads (*n* = 5 each) (Fig. 3l). Moreover, premature anagen was activated by P54 in *Tyr-rtTA*;*tetO-Spp1* mice, induced with doxycycline starting from P42. Compared with doxycycline-treated control mice, which remained in telogen, *Tyr-rtTA*;*tetO-Spp1* mice displayed broad anagen activation (*n* = 3 mice each) (Fig. 3m,n). Therefore, SPP1 is sufficient to induce new hair growth and it mediates hair growth activation in at least two skin states: melanocytic nevus and wound healing.

## CD44 mediates the osteopontin effect

**Extended Data Fig. 9:**
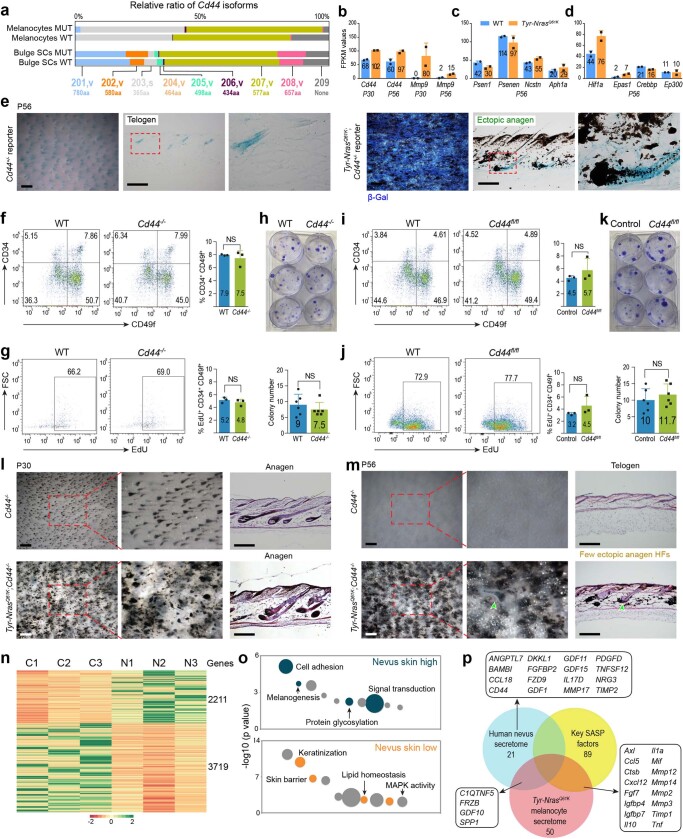
Expression and the effect of *Cd44* deletion on hair growth. **a-e**, Expression of *Cd44* and related genes. **a**, Relative abundance of *Cd44* isoforms established from full-length bulk RNA-seq. Isoforms are numbered using conventional nomenclature and indicated along the X-axis. *Cd44v* isoforms are designated with “v” and *Cd44s* isoform with “s”. Skin cell types are listed along the Y-axis. WT – wild type cells, MUT – *Tyr-Nras*^*Q61K*^ mutant cells. Bulge stem cells are enriched for *Cd44v* isoforms 201, 202, 205 and 208. **b**, *Cd44* is prominently expressed on bulk RNA-seq in bulge stem cells from WT control (blue) and *Tyr-Nras*^*Q61K*^ mice (orange) both at P30 and P56. *Mmp9*, direct downstream target of CD44-ICD signalling is prominently overexpressed in *Tyr-Nras*^*Q61K*^ bulge stem cells both at P30 and P56. **c**, Expression values of γ-secretase complex genes in P56 WT and *Tyr-Nras*^*Q61K*^ bulge stem cells. **d**, Expression values of transcription factors mediating CD44-ICD signalling in P56 WT and *Tyr-Nras*^*Q61K*^ bulge stem cells. Average FPKM values are shown on **b–d**. In **b-d**, *n* = 2. **e**, LacZ staining (blue) in *Tyr-Nras*^*Q61K*^*;Cd44*^*+/−*^ *vs*. control *Cd44*^*+/−*^ P56 reporter mice showed LacZ^+^ cells in the skin, both in epithelial and dermal compartments. For each panel, wholemount and histology samples are shown on the left and on the right, respectively. **f-m**, Effects of *Cd44* deletion on bulge stem cells and hair cycle status in nevus mice. **f**, Total abundance of CD34^+^/CD49f^+^ bulge stem cells remained unchanged in germline *Cd44*^*-/-*^ mice *vs*. WT control mice. In **f**, *n* = 3; *P* = 0.52. **g**, Labeling efficiency of bulge stem cells after 7 days of EdU pulse remained unchanged in germline *Cd44*^*-/-*^ mice *vs*. WT control mice. In **g**, *n* = 3; *P* = 0.401. In (**f, g**) representative cytometry plots are shown on the left, and data quantification on the right. **h**, In *in vitro* culture assay on FACS-isolated bulge stem cells, clonogenic potential of CD34^+^/CD49f^+^ cells remained unchanged in germline *Cd44*^*−/−*^ mice *vs*. WT control mice. Top – representative culture plates, bottom – data quantification. In **h**, *n* = 6; *P* = 0.384. **i**, Total abundance of CD34^+^/CD49f^+^ bulge stem cells remained unchanged in epithelial-specific conditional *K14-Cre;Cd44*^*fl/fl*^ (aka *Cd44*^*fl/fl*^) mice *vs*. control mice. In **i**, *n* = 3; *P* = 0.328. **j**, Labeling efficiency of bulge stem cells after 7 days of EdU pulse remained unchanged in *Cd44*^*fl/fl*^ mice *vs*. control mice. In **j**, *n* = 3; *P* = 0.218. In (**i, j**) representative cytometry plots are shown on the left, and data quantification on the right. **k**, In *in vitro* culture assay on FACS-isolated bulge stem cells, clonogenic potential of CD34^+^/CD49f^+^ cells remained unchanged in *Cd44*^*fl/fl*^ mice *vs*. control mice. Top – representative culture plates, bottom – data quantification. In **k**, *n* = 6; *P* = 0.411. **l, m**, *Tyr-Nras*^*Q61K*^*;Cd44*^*−/−*^ mice showed rescue of hair cycle quiescence. Unlike *Tyr-Nras*^*Q61K*^ mice (see Extended Data Fig. 1), *Tyr-Nras*^*Q61K*^*;Cd44*^*−/−*^ animals showed synchronized anagen at P30 (**l**) and only very occasional ectopic anagen HFs at P56 (**m**). For both time points, representative *Cd44*^*−/−*^ control and *Tyr-Nras*^*Q61K*^*;Cd44*^*−/−*^ mutant skin samples are shown. Wholemount samples are shown on the right and histology on the left of each panel. **n–o**, Bulk RNA-seq between hairy nevi and adjacent normal facial skin in humans. DEGs heatmap is shown in (**n**), bubble chart with enriched (blue) and depleted (orange) GO terms in human nevi is shown in (**o**). **p**, Comparisons of human nevus secretome (blue) with *Tyr-Nras*^*Q61K*^ mouse melanocyte secretome (red) and published in vitro SASP (yellow). In **b–d, f–k**, *n* = independent experiments. Data are mean ± s.d. *P* values are calculated using unpaired two-tailed Student’s t-test. NS, *P* ≥ 0.05. Scale bars, **e, l, m** (wholemount) – 500 μm; **e, l, m** (histology) – 200 μm. Source data

SPP1 signals via distinct binding sites to its cognate receptors: β-integrins and CD44 (also known as CSPG8). Of these, CD44 is an established stemness marker in several cancer types, where it promotes proliferation, invasiveness and radio-resistance^28^. SPP1 preferentially binds to alternatively spliced CD44v isoforms, which show enrichment in bulge SCs on RNA-seq (Extended Data Fig. 9a). In response to SPP1, CD44 undergoes proteolytic cleavage by γ-secretase, which releases its nuclear-targeted intracellular domain (CD44-ICD), thus coactivating HIF1A, EPAS1, EP300 and CREBBP to regulate gene expression^28^. *Mmp9*, a direct downstream target of CD44-ICD signalling^29^, is one of the top upregulated genes in *Tyr-Nras*^*Q61K*^ bulge SCs (Extended Data Figs. 6c and 9b) and bulge SCs retain high expression of all γ-secretase subunits as well as CD44-ICD-binding transcriptional factors (Extended Data Fig. 9c,d).

**Fig. 4:**
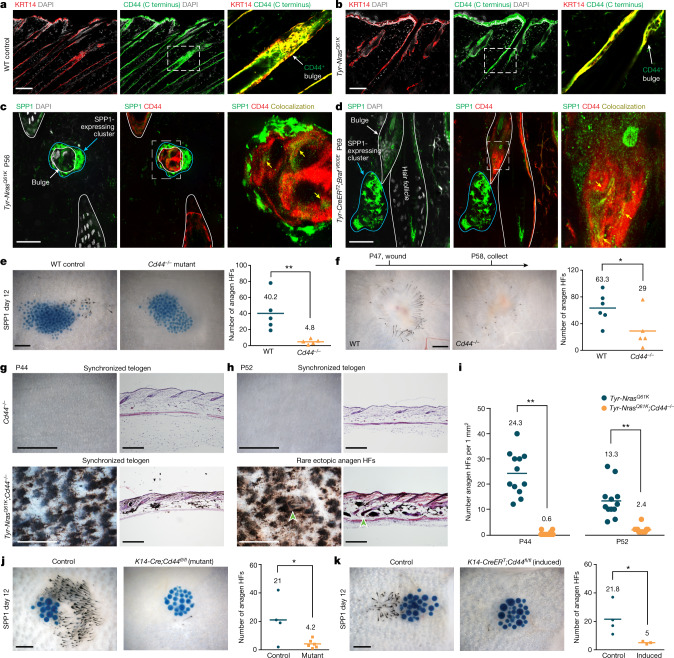
Effect of SPP1 on hair growth depends on CD44. **a**,**b**, Epithelial HF cells in both WT control (**a**) and *Tyr-Nras*^*Q61K*^ (**b**) mice strongly expressed CD44. Samples were also stained for the epithelial keratin marker KRT14. **c**,**d**, Co-staining for SPP1 and CD44 in *Tyr-Nras*^*Q61K*^ (**c**) and *Tyr-CreER*^*T2*^;*Braf*^*V600E*^ (**d**) skin revealed SPP1^high^ clusters of dermal cells adjacent to CD44^+^ bulge cells with weaker colocalizing SPP1 signal (yellow arrows). **e**, *Cd44*^−/−^ mice showed significantly reduced anagen activation in response to SPP1-soaked beads compared with WT mice. Representative samples (left) and quantification (right) are displayed. *n* = 5; *P* = 0.00938. **f**, *Cd44*^−/−^ mice showed reduced wound-induced hair growth compared with WT mice. Representative samples (left) and quantification (right) are displayed. *n* = 6 in WT and *n* = 5 in *Cd44*^−/−^; *P* = 0.0494. **g**,**h**, *Tyr-Nras*^*Q61K*^;*CD44*^−/−^ mice lacking *Cd44* showed rescue of hair cycle quiescence. At P44, *Tyr-Nras*^*Q61K*^;*Cd44*^−/−^ HFs were in coordinated telogen (**g**). Only rare anagen HFs (arrowheads) were present at P52 (**h**). **i**, Quantification of anagen HFs in *Tyr-Nras*^*Q61K*^ versus *Tyr-Nras*^*Q61K*^;*Cd44*^*−/−*^ mice. Double mutants showed reduced ectopic anagen at P44 and P52. At P44, *n* = 12 and *P* = 0.00000000249; at P56, *n* = 12 and *P* = 0.0000166. **j**,**k**, Both constitutive epithelial-specific *K14-Cre*;*Cd44*^*fl/fl*^ (**j**) and tamoxifen-induced *K14-CreER*^*T*^;*Cd44*^*fl/fl*^ (**k**) mice showed significantly reduced anagen activation in response to SPP1-soaked beads compared with control mice. Representative samples (left) and quantification (right) are displayed. In **j**, *n* = 4 in control and *n* = 6 in mutant; *P* = 0.0352. In **k**, *n* = 4 in control and *n* = 3 in induced mutant; *P* = 0.0476. In **e**,**f**,**i**–**k**, *n* refers to biologically independent samples. *P* values are calculated using unpaired two-tailed Student’s *t*-test. *P ≤ 0.05 and **P ≤ 0.01. Scale bars, 50 μm (**c**,**d**), 100 μm (**a**,**b**), 200 μm (histology; **g**,**h**), 300 μm (**j**,**k**), 500 μm (**e**,**f**) and 1 mm (wholemount; **g**,**h**). Source data

We asked whether CD44 mediates hair growth hyperactivation in the nevus. Consistent with previous single-cell RNA-seq profiling, CD44 is prominently expressed across all epithelial compartments of the HF^27^, including in bulge SCs, both in control and *Tyr-Nras*^*Q61K*^ mice (Fig. 4a and Extended Data Fig. 9e). At the protein level, SPP1 colocalizes with CD44 in bulge SCs in both *Tyr-Nras*^*Q61K*^ and *Tyr-CreER*^*T2*^;*Braf*^*V600E*^ mice (Fig. 4c,d). Next, we asked whether *Cd44* deletion compromises bulge SC abundance and proliferative potential. The percentage of either total CD34^+^CD49f^+^ bulge SCs or their EdU-labelled subset after 7 days of EdU pulse did not significantly change in germline *Cd44*^−/−^ mutant versus control mice (*n* = 3 each) (Extended Data Fig. 9f,g) as well as in epithelial-specific constitutive *K14-Cre*;*Cd44*^*fl/fl*^ mutant versus control mice (*n* = 3 each) (Extended Data Fig. 9i,j). Also unchanged was the in vitro colony-forming potential by sorted bulge SCs both from *Cd44*^−^^*/*−^ and *K14-Cre*;*Cd44*^*fl/fl*^ mice versus control animals (*n* = 6 each) (Extended Data Fig. 9h,k). Therefore, loss of CD44 alone does not compromise key bulge SC properties. Next, we asked whether CD44 function is required for HF response to SPP1. Indeed, anagen induction in response to SPP1-soaked beads was significantly suppressed in *Cd44*^−^^/−^ versus control mice (*n* = 5 each) (Fig. 4e). Likewise, significantly fewer anagen HFs were induced at the wound margin of *Cd44*^−^^/−^ mutant (*n* = 5) versus control mice (*n* = 6) (Fig. 4f). Furthermore, *Cd44* deletion in *Tyr-Nras*^*Q61K*^;*Cd44*^−^^/−^ mice led to rescue of ectopic hair cycling, phenocopying the effect of *Spp1* deletion in the *Tyr-Nras*^*Q61K*^ background (Fig. 4g–i and Extended Data Fig. 9l,m). Loss of SPP1 responsiveness in the soaked bead experiment was also phenocopied upon epithelial-specific *Cd44* deletion in *K14-Cre*;*Cd44*^*fl/fl*^ as well as in tamoxifen-inducible *K14-CreER*^*T*^;*Cd44*^*fl/fl*^ mice. Compared with SPP1-treated control mice (*n* = 4 each), the numbers of induced anagen HFs were significantly reduced both in *K14-Cre*;*Cd44*^*fl/fl*^ (*n* = 6) (Fig. 4j) and in induced *K14-CreER*^*T*^;*Cd44*^*fl/fl*^ (*n* = 3) mice (Fig. 4k). Therefore, the hair growth-activating effect of SPP1 in nevus skin requires epithelial CD44 signalling.

## Human hairy nevi upregulate osteopontin

**Fig. 5:**
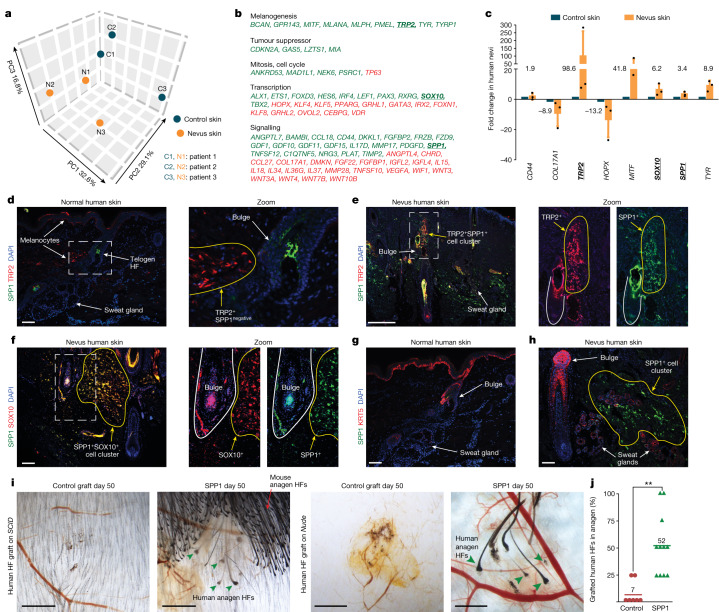
Human nevi feature secretome enriched for SPP1. **a**, Bulk RNA-seq reveals prominent differences between hairy nevi and adjacent normal facial skin in humans. A principal component analysis plot is shown. See Extended Data Fig. 9. **b**, Selected upregulated (by 2× or more; green) and downregulated (by 2× or more; red) differentially expressed genes in nevus versus normal human skin. Bold and underlined genes were validated by qRT-PCR. **c**, qRT–PCR of selected differentially expressed genes from bulk RNA-seq data. *n* = 3. **d**,**e**, SPP1 and TRP2 co-staining. In normal skin, TRP2^+^ melanocytes did not express SPP1 (**d**), whereas in nevus skin, clusters of TRP2^+^SPP1^+^ cells were seen next to HF bulge regions (**e**). **f**, SPP1 and SOX10 co-staining. Nevus skin contained SOX10^+^SPP1^+^ cell clusters next to HF bulge regions. **g**,**h**, SPP1 and KRT5 co-staining. Unlike in normal skin (**g**), SPP1^+^ cell clusters were seen next to HFs in nevus human skin (**h**). **i**,**j**, SPP1 microinjections induced precocious growth by human scalp HFs (arrowheads). Representative samples of human HFs on day 50 post-grafting (**i**) and quantification of human HFs in anagen (**j**) are shown. In **j**, *n* = 7 for control and *n* = 11 for SPP1; *P* = 0.00034. In **c**, *n* refers to independent experiments. In **j**, *n* refers to biologically independent samples. *P* values were calculated using unpaired two-tailed Student’s *t*-test. **P ≤ 0.01. Scale bars, 100 μm (**d**–**h**) and 1 mm (**i**). Source data

We also examined signalling aspects of congenital hairy nevi in humans. Whole-tissue RNA-seq revealed prominent differences between congenital hairy nevi and adjacent normal facial skin, and patient-to-patient variability (Fig. 5a, Extended Data Fig. 9n,o and Supplementary Table 5). Nevi showed enrichment for the melanogenesis genes *BCAN*, *GPR143*, *MITF*, *MLANA*, *MLPH*, *PMEL*, *SOX10*, *TRP2*, *TYR* and *TYRP1*, and consistent with *Tyr-Nras*^*Q61K*^ mouse data, they upregulated the tumour suppressor genes *CDKN2A*, *GAS5*, *LZTS1*, *MIA* and the mitotic markers *ANKRD53*, *MAD1L1*, *NEK6* and *PSRC1*, albeit the latter can be contributed by proliferating HF cells. Among secreted factors, nevi upregulated *SPP1*, several TGFβ/BMP members *GDF1/10/11/15* and *BAMBI*, the WNT modulators *DKKL1*, *FRZB,* as well as *CCL18*, *IL17D* and *PDGFD* (Fig. 5b). *SPP1* was among upregulated secretome factors shared between human hairy nevi consistently across patients and *Tyr-Nras*^*Q61K*^ mouse melanocytes (Extended Data Fig. 9p), which we validated by quantitative PCR with reverse transcription (qRT–PCR) (Fig. 5c) and immunostaining (Fig. 5d–h). SPP1 expression was prominent in dermal clusters of either TRP2^+^ (Fig. 5e) or SOX10^+^ melanocytes (Fig. 5f) surrounding bulge regions of HFs. Finally, we tested the hair growth-inducing effect of SPP1 on human scalp HFs in albino *Nude* or pigmented *SCID* host mice^8^. Skin next to telogen HFs were treated with three daily doses of SPP1 or saline. Compared with control (*n* = 7), SPP1 (*n* = 11) accelerated anagen entry in human HFs, sometime accompanied by anagen entry in mouse HFs (Fig. 5i,j). We conclude that SPP1 is a nevus melanocyte-derived hair growth activator in humans.

## Discussion

In this work, we studied how melanocytic skin nevi develop hair overgrowth, which led us to discover that senescent cells can prominently activate tissue-resident SCs and stimulate regeneration. Traditionally, accumulation of senescent cells in tissues is viewed as detrimental to their regenerative potential. This scenario plays out during natural advanced ageing, pathologically accelerated ageing or upon genotoxic exposure^4^. Broad build-up of senescent cells depletes the regenerative capacity of tissues in part via direct elimination of SCs (that is, many SCs become senescent and, thus, non-proliferative) and in part via excessive activation of cytokine-rich secretome (that is, SASP)^17^. SASP factors induce a state akin to low-grade inflammation, which, when persistent, triggers tissue fibrosis. Not surprisingly, systemic depletion of senescent cells in mice delays ageing phenotypes^30^, whereas senolytics, drugs that selectively kill senescent cells, have emerged as promising candidate therapeutics for age-related pathologies^31^.

**Extended Data Fig. 10:**
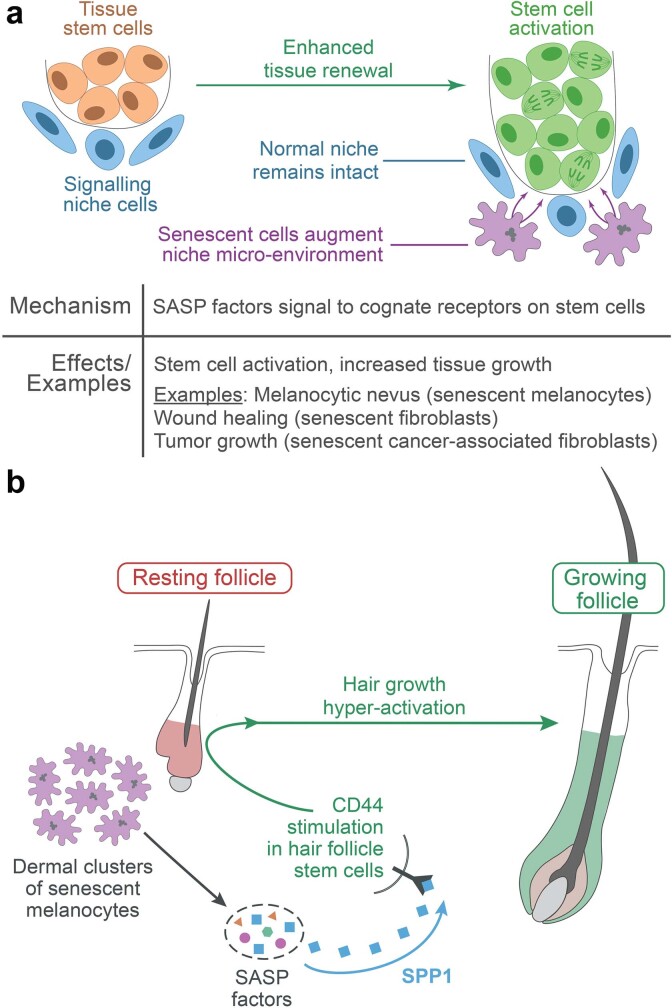
Model of senescent cell-induced hair growth in skin nevus. **a**, Limited accumulation of senescent cells adjacent to normal, intact stem cell niche can augment it and result in stem cell activation. Mechanism, effects, and examples are listed below the schematic drawing. Tissue stem cells – orange (quiescent) and green (activated); normal niche cells – blue; senescent cells – purple. **b**, Schematic representation of the mechanism driving hair growth hyperactivation in skin nevus. Dermal clusters of senescent melanocytes (purple) secrete SASP factors (colored geometric shapes). SPP1 (blue squares) is the leading SASP factor of senescent melanocytes. It signals via CD44 receptor (black Y shape) to epithelial stem cells in adjacent hair follicles, inducing them into precocious growth (green arrow).

However, recent evidence points towards alternative, beneficial effects of senescent cells on tissue growth. Senescent cells form in multiple embryonic tissues, including in the apical ectodermal ridge of the developing limb in mice^18^. Such ‘developmental’ senescent cells secrete signalling factors thought to instruct growth by surrounding non-senescent embryonic cells. Senescent cells also frequently emerge in non-aged adult tissues upon injury, where SASP factors stimulate enhanced repair. This scenario has been observed in zebrafish after fin amputation^32^, in mice following exercise-induced or cardiotoxin-induced skeletal muscle injury^33,34^, surgical resection of liver^35^ and excisional skin wounding^20^. In tumours, excessive growth by cancer-initiating cells can rely on stimulating paracrine signals from adjacent senescent cells. The latter can form among cancer-associated stromal cells^21,22^ or within cancer cell lineage itself, either triggered by an oncogenic mutation (OIS mechanism) or genotoxic anticancer therapy (DNA damage-induced senescence)^36^. The above examples teach that the paracrine component of the cellular senescence program is commonly used as part of the tissue growth-promoting mechanism. The mechanism of hair overgrowth reported by us in skin nevi exemplifies growth-promoting property of senescent cells (Extended Data Fig. 10). In Supplementary Discussion 1, we discuss conditions necessary for the promoting effect of senescent cells on tissue growth and insights offered by the hairy nevus model.

Whether hairy melanocytic nevus is an outlying example of the kind of effects that senescent cells exert on HFs still remains unknown. Indeed, commonly reduced rather than enhanced hair growth is observed in animal models and in people with increased senescent cell burden—advanced age, progeria or exposure to radiation and chemotherapy. Hair overgrowth is also a leading clinical presentation of smooth muscle hamartoma, a congenital or acquired benign nevus-like condition driven by OIS-activating mutations in cutaneous smooth muscle cells^37^. At the same time, nevus sebaceous, where keratinocytes carry OIS-activating mutations, does not present hair overgrowth, but instead features exuberantly enlarged sebaceous glands^38^. We posit that the exact tissue-level consequence of senescence (for example, hair growth versus sebaceous hypertrophy) depends on the exact molecular composition of SASP, which in turn depends on the original lineage of cells that become senescent, the senescence-inducing mechanism and possibly other factors. That SASP composition is probably heterogenous is also strongly supported by molecular data emerging from other recent studies on the senescent cell secretome (reviewed in Supplementary Discussion 2).

SPP1 is the lead SASP factor secreted by senescent dermal melanocytes that potently induces hair growth. SPP1 is also the topmost SASP factor produced by senescent cancer-associated fibroblasts^22^, and its signalling via CD44 promotes cancer cell stemness, tumour growth and radio-resistance^28^. We showed that the hair growth-promoting effect of SPP1 also requires an intact CD44 receptor on epithelial cells. Consistently, an SPP1 sequence-based synthetic peptide lacking the CD44-binding site fails to promote epithelial proliferation in cultured human HFs^39^. In this context, our data points to future hair growth-stimulating therapies in which select SASP factors, such as SPP1 or its CD44-binding derivatives, are injected into hair loss-affected skin. In support of this approach are clinical cases reporting hair loss-resistant melanocytic nevi on the scalp of patients with alopecia, including alopecia universalis^40^.

Several intriguing questions arise from our study that require future investigation. First, not all melanocytic nevi in people are hairy, probably because they do not satisfy all of the conditions necessary for the growth-promoting effect of senescent cells. In-depth comparison of hairy versus non-hairy human nevi will probably reveal new cellular and molecular diversity of these understudied tissue states. Second, in addition to growing more frequently, hairs in human nevi also become thicker and longer, a property known as terminalization. Because hairs in mice cannot undergo terminalization, future studies on human nevus hairs will probably reveal additional signalling effects of SASP on HF cells, beyond SCs. Third, despite carrying activating oncogene mutations, melanocytes in both *Tyr-Nras*^*Q61K*^ and Tyr-*CreER*^*T2*^;*Braf*^*V600E*^ mice become senescent in the dermis next to HFs, but not within HFs themselves. This suggests that a distinct signalling microenvironment within HFs can effectively counteract the OIS mechanism. Future studies comparing signals that melanocytes receive from other cells in their dermal versus HF locations will probably identify new senescence-preventing pathways. Last, normally, melanocytes are not critical regulators of HF SCs and hair growth timing (that is, grey hairs still grow robustly). Thus, acquisition of senescence can confer non-niche cells with novel niche-like properties. By the same accord, acquisition of senescence and SASP by ‘professional’ niche cells (for example, dermal papilla fibroblasts in HFs) may endow them with new regulatory properties. Future works should seek similar effects of cellular senescence on SC functions in other actively renewing organs, such as gut and bone marrow.

In conclusion, our study into the peculiar, yet poorly understood skin condition of hairy nevus led us to identify a distinct regulatory mechanism for adult SCs by tissue-resident senescent cells. These findings have far-reaching implications for advancing our understanding of SC niche regulation and for developing new therapeutic strategies to regenerative disorders.

## Methods

### Experimental mouse models

The following mouse lines were used: *Tyr-Nras*^*Q61K*^, *Tyr-rtTA*, *Tyr-CreER*^*T2*^, *Tyr(C-2J)*, *Braf*^*V600E*^, *Trp53*^*flox*^, *Spp1*^−/−^, *Spp1*^*flox*^, *tetO-Spp1*, *Cd44*^−/−^, *Cd44*^*flox*^, *K14-Cre*, *K14-CreER*^*T*^, *K14-H2B-GFP*, *K14-Edn3*, *K14-Kitl*, *tdTomato*, *TOPGAL*, *Nude* and *SCID*. Tissue-specific mouse models were produced by crossing either *Cre*-carrying or *CreER-*carrying animals with *flox-ed* gene carrying animals, or *rtTA*-carrying animals with *tetO*-carrying animals. All animal experiments followed all relevant guidelines and regulations and were approved by the Institutional Animal Care and Use Committee at China Agricultural University (to Z.Y.) and/or the Animal Care Committee at Gifu University (to T.K.) and/or the Animal Care and Use Committee of National Taiwan University (to C.-H.K.) and/or the Institutional Animal Care and Use Committee at University of California, Irvine (to B.A. and/or A.K.G. and/or M.V.P.) and/or the Institutional Animal Care and Use Committee at Central South University (to J.L.) and/or the Institutional Animal Care and Use Committee at Kyungpook National University (to J.W.O.).

### Mouse induction protocols

Tetracycline-controlled overexpression of SPP1 in melanocytes was achieved in *Tyr-rtTA*;*tetO-Spp1* mice with 2 mg ml^−1^ doxycycline hyclate (Sigma) in 5% sucrose and a doxycycline-containing diet (Bio-Serv, 200 mg kg^−1^) provided ad libitum. Inducible conditional gene recombination was achieved in *CreER*-carrying and *flox-ed* gene-carrying animals by intraperitoneal injection of tamoxifen (Sigma) in corn oil at a dose of 75 mg kg^−1^. In P2 animals, inducible conditional gene recombination was achieved by topical administration of (Z)-4-hydroxytamoxifen (4-HT; Sigma) in DMSO at 75 mg ml^−1^.

### EdU pulse and pulse-chase assays

Mice were intraperitoneally injected with EdU (5 µg g^−1^ body weight) daily for seven consecutive days (pulse period), followed by an 8-week chase period. A portion of harvested skin was examined histologically using an EdU imaging kit (Thermo Fisher). Remaining skin portion was used to isolate cells for flow cytometry-based quantification using an EdU flow kit (Thermo Fisher). Triple-positive CD34^+^CD49f^+^EdU^+^ cells were used to quantify EdU^+^ bulge SCs.

### Protein injection procedure

Intradermal delivery of protein-soaked agarose beads was performed as previously described^8,11^. In brief, recombinant SPP1 protein (441-OP, R&D) was reconstituted in 0.1% BSA to a final concentration of 1.3 mg ml^−1^. Affi-gel blue beads (Bio-Rad) were washed three times in sterile PBS, air dried and resuspended in reconstituted recombinant protein solution. Beads were incubated on ice for 1 h before implantation. For both recombinant protein and BSA controls, beads were implanted intradermally in P51–P53 animals. Bead implantation sites were resupplied with additional protein at 24, 48 and 72 h.

### Skin wounding procedure

Mice were shaved and skin was cleaned with antiseptic. Surgery was conducted under continuous isoflurane anaesthesia. A full-thickness excisional wound was created without injuring the underlying fascia with dermal biopsy punch. Mice were given post-surgical analgesia: subcutaneous ketoprofen, followed by acetaminophen in drinking water.

### Flow cytometry and FACS procedures

Dorsal skin was digested into single cells with Dispase II solution (Roche), followed by collagenase I solution (Life Technologies). Cells were filtered first through 70-µM and then 40-µM strainers. Viability dye (BioLegend) was used to exclude dead cells. Cell suspension was stained with primary antibodies in FACS staining buffer (1% BSA in PBS with 2 mM EDTA) for 30 min on ice before sorting. The following antibodies were used: mouse anti-γH2AX (1:100; 564718, BD Biosciences), mouse anti-TRP2 (1:50; sc-74439 AF647, Santa Cruz Biotechnology), rat anti-Ki67 (1:50; 58-5698-82, Thermo Fisher), rat anti-CD117 (1:100; 105812, BioLegend), rat anti-CD45 (1:50; 103108, BioLegend), rat anti-CD34 (1:50; 560230, BD Biosciences), rat anti-CD49f (1:100; 555736, BD Biosciences) and rabbit anti-SPP1 (1:100; 702184, Thermo Fisher). Cells were sorted on FACSAria II sorters (BD Biosciences) and flow cytometry analysis was performed on LSRII flow cytometer (BD Biosciences). Data were analysed with FlowJo software (version 10.8.0). Expression of SPP1 protein was detected using staining of both permeabilized cells (permeabilized condition) and non-permeabilized cells (surface-bound condition). Under permeabilized condition, we measured total SPP1 present in cells, whereas under surface-bound conditions, we measured SPP1 present on the cell surface, such as bound to its receptors. For permeabilization, cells were washed in PBS and resuspended at 1 million cells per 100 μl, permeabilization buffer was added and cells were stained following Fixation/Permeabilization kit instructions (BD Biosciences).

### Primary melanocyte culture assay

Melanocytes were purified from P0 mouse skin by FACS as CD117^+^CD45^neg^ populations. Sorted cells were then cultured in complete primary melanocyte media (RPMI 1640, 5% FBS, antibiotic–antimycotic, 2.5 ng l^−1^ basic human fibroblast growth factor, 10 μM ethanolamine, 1 mg ml^−1^ insulin, 1 μM *O*-phosphoethanolamine, 5 nM endothelin, 25 nM α-MSH and 50 ng ml^−1^ murine SC factor) at 37 °C with 5% CO_2_.

### H_2_O_2_ treatment procedure

Cultured melanocytes in culture dishes or chamber slides were treated with H_2_O_2_ (Sigma) at 100 mM or vehicle (medium 254 and HMGS-2) for 2 h at 37 °C. Treated cells were rinsed twice with PBS.

### DiI labelling procedure

Cells were labelled with DiI dye (Thermo Fisher) following the manufacturer’s instructions. In brief, cells were incubated for 15 min at 37 °C in culture medium supplied with 5 µl of the cell-labelling solution per 1 ml. After labelling, cells were dissociated with Accutase (Stemcell Technologies), followed by two washes with PBS.

### Cell injection procedure

Cells were counted using a haemocytometer and then diluted to 2,000 cells per microlitre in cell culture medium. Of cell suspension, 10–50 µl was slowly injected intradermally into the dorsal skin of recipient mice using a 29-G needle.

### Grafting procedure

Skin micro-grafts containing four to six anagen HFs were transplanted to the dorsal skin of 6-to-8-week-old female *SCID* or *Nude* mice, as previously described^8^. Thirty days post-grafting, 10 µl of recombinant protein or saline was microinjected to the HF grafting site for 3 consecutive days. Host mice were euthanized on post-grafting day 50 and skin was analysed on wholemount.

### ABT-737 treatment procedure

Mice were subcutaneously injected twice (on days P10 and P12) with ABT-737 (Cayman Chemical) or vehicle control at a dose of 75 mg kg^−1^.

### β-Gal staining

For β-galactosidase staining, thick sections (20 µm) were incubated in 1 mg ml^−1^ X-gal substrate in PBS with 1.3 mM MgCl_2_, 3 mM K_3_Fe(CN)_6_ and 3 mM K_4_Fe(CN)_6_ at 37 °C overnight. For senescence-associated β-gal staining, cells were stained using a kit (Cell Signaling) according to the manufacturer’s instructions. In brief, cells were fixed with fixative solution provided by the manufacturer for 15 min at room temperature, followed by acidic β-gal detection using pH 6.0 staining solution overnight at 37 °C.

### Immunohistochemical staining

For paraffin-embedded sections, skin samples were fixed with 4% (vol/vol) paraformaldehyde overnight at 4 °C. Histological sections were permeabilized for 15 min in PBS + 0.1% Triton X-100 (PBST) and blocked for at least 1 h at room temperature with PBST + 3% BSA. Mouse antibodies were blocked with the M.O.M. block kit (Vector Laboratories). Primary antibodies were incubated overnight at 4 °C and secondary antibodies were incubated for 1 h at room temperature. The following primary antibodies were used: rabbit anti-γH2AX (1:300; 9718, Cell Signaling), rabbit anti-TRP2 (1:200; ab74073, Abcam), rabbit anti-TRP2 (1:200; ab103463, Abcam), mouse anti-PCNA (1:1,000; ab29, Abcam), rat anti-CD34 (1:100; 14-0341-82, Thermo Fisher), rabbit anti-SOX9 (1:200; AB5535, Millipore), goat anti-SPP1 (1:100; AF808, R&D), goat anti-SPP1 (1:300; AF1433, R&D), rabbit anti-KRT14 (1:2,000; ab119695, Abcam), rabbit anti-CD44 (1:100; PA5-94934, Thermo Fisher), rabbit anti-SOX10 (1:100; ab180862, Abcam), rabbit anti-KRT5 (1:1,000; 905501, BioLegend) and goat anti-Pcad (1:200; AF761, R&D Systems). The following secondary antibodies were used: donkey anti-rat AF555 (1:1,000; ab150154, Abcam), donkey anti-rabbit AF555 (1:1,000; A31572, Thermo Fisher), donkey anti-mouse AF555 (1:1,000; A31570, Thermo Fisher), donkey anti-rabbit AF488 (1:1,000; A21206, Thermo Fisher), donkey anti-goat AF488 (1:1,000; A11055, Thermo Fisher), goat anti-rat AF488 (1:1,000; A11006, Thermo Fisher), goat anti-rabbit AF488 (1:1,000; 4412s, Cell Signaling), goat anti-mouse AF555 (1:1,000; 4409s, Cell Signaling) and goat anti-rabbit AF555 (1:1,000; 4413s, Cell Signaling).

### RNAscope staining

RNA staining was performed using the Multiplex Fluorescent v2 kit (Advanced Cell Diagnostics). In brief, skin was frozen in OCT compound and sectioned at 12–15 µm. Sections were fixed at room temperature for 1 h with 4% paraformaldehyde in PBS, followed by standard manufacturer’s protocols (Advanced Cell Diagnostics). RNA probes for hybridization were purchased from Advanced Cell Diagnostics and included *Mm-Spp1* (catalogue no. 435191-C1), *Mm-Dct-C2* (*Trp2*; 460461-C2), *Mm-Cdkn2b* (*p15*; 458341-C1), *Mm-Cdkn2a* (*p16*; 411011-C1), *Mm-Mki67-C3* (*Ki67*; 416771-C3) and *Mm-Aurkb* (461761-C1).

### Western blot assay

Single sorted melanocytes or cells from mouse whole-back skin were lysed in RIPA buffer (Sigma) containing a cocktail of protease inhibitors (Thermo Fisher). Of each cell lysate, 25 µg was loaded onto a 12% separating Bis-Tris gel. Proteins were transferred to a nitrocellulose membrane. Membrane was incubated with primary goat anti-mouse SPP1 antibody (1:100; AF808, R&D) or rabbit anti-β-actin antibody (1:1,000; 4967, Cell Signaling) at a concentration of 2.5 μg ml^−1^. The blot was developed with Enhanced Chemiluminescence Plus Developer (Fisher Scientific).

### ELISA

SPP1 levels in the supernatant of cell cultures were measured by a mouse OPN/SPP1 ELISA kit (Thermo Fisher) according to the manufacturer’s instructions. In brief, SPP1 concentration was calculated by generating a standard curve from recombinant SPP1 protein diluted between 0 and 2,000 pg ml^−1^. Microplates were measured using a Synergy microplate reader (BIO-TEK) at a wavelength of 450 nm.

### Real-time PCR assay

Total RNA from sorted cells was extracted using RNeasy Micro Kit (Qiagen) coupled with its on-column DNase digestion protocol. Total RNA was then reverse-transcribed with Superscript III (Life Technologies) in the presence of oligo-dT. Full-length cDNA was normalized to an equal amount using housekeeping genes *GAPDH* or 18S. Primers are listed in Supplementary Table 6.

### Colony-forming assay

Sorted GFP-expressing HF bulge SCs and hair germ progenitors from *K14-H2B-GFP* mice were plated onto 3T3 fibroblast feeder layer cells, pre-treated with mitomycin C to induce cell cycle arrest. Cells were co-cultured at 37 °C in William’s E medium supplemented with calcium and antibiotic–antimycotic. Medium was replaced after 48 h, and the attachment rate was evaluated following an additional 12 h of culture. Attached cells were passaged upon confluence, which was achieved every 4–6 days. Calcium-supplemented culture medium was changed every 2–3 days. In other experiments, bulge SCs were FACS sorted as CD34^+^CD49f^+^ cells and cultured at a concentration of 1,000 cells per squared centimetre, in the presence of mitomycin C inactivated 3T3 fibroblasts. After 2 weeks, 0.5% crystal violet (Sigma) solution made in 1:1 ratio of water:methanol was added to each culture well. Stained plates were then rinsed with water, air dried and imaged.

### Human skin samples

Collection of human skin samples followed all relevant guidelines and regulations and was approved by the Research Ethics Committee at National Taiwan University Hospital and/or the Medical Ethics Committee at Kyungpook National University Hospital and/or the Ethics Committee of Xiangya Hospital, Central South University and comply with guidelines from the Ministry of Science and Technology (MOST) of the People’s Republic of China. All participants provided written informed consent. No identifiable images of human research participants are shown.

### Bulk and single-cell RNA-seq for mouse tissue

For bulk RNA-seq, total RNA was extracted from FACS-sorted cells in biological triplicates with an RNA integrity number of more than 9.1, and 1 ng of mRNA was used for full-length cDNA synthesis, followed by PCR amplification using Smart-seq2. The libraries were sequenced on the Illumina Next-Seq500 system to an average depth of 10–30 million reads per library using paired 43-bp reads.

For single-cell RNA-seq, cells were captured using the Fluidigm C1 chips as per the manufacturer’s protocol. A concentration of 200,000–350,000 cells per millilitre was used for chip loading. After cell capture, chips were examined visually under the microscope to determine the capture rate, and empty chambers or chambers with multiple cells were excluded from the analysis. cDNA was synthesized and amplified on the Fluidigm C1 Single-Cell Auto Prep System with the Clontech SMARTer Ultra Low RNA kit and the ADVANTAGE-2 PCR kit (Clontech). Single-cell RNA-seq libraries were constructed in 96-well plates according to the Fluidigm C1 manual. Multiplexed libraries were analysed on Agilent 2100 Bioanalyzer for fragment distribution and quantified using Kapa Biosystem’s universal library quantification kit. Libraries were sequenced as 75-bp paired-end reads on the Illumina Next-Seq500 platform.

For both bulk and single-cell RNA-seq, reads were first aligned using STAR v.2.4.2a with parameters ‘--outFilterMismatchNmax 10 --outFilterMismatchNoverReadLmax 0.07 --outFilterMultimapNmax 10’ to the reference mouse genome (mm10/genocode,vM8). Gene expression levels were quantified using RSEM v.1.2.25 with expression values normalized into fragments per kilobase of transcript per million mapped reads (FPKM). Samples with more than 1,000,000 uniquely mapped reads and more than 60% uniquely mapping efficiency were used for downstream analyses. Differential expression analysis was performed using edgeR v.3.2.2 on protein-coding genes and long non-coding RNAs. Differentially expressed genes were selected by using fold change ≥ 2, false discovery rate < 0.05 and counts per million reads ≥ 2.

### Bulk RNA-seq for human tissue

RNA was extracted from human hairy nevus skin as well as normal skin from nevus edge using the Qiagen RNA extraction kit. cDNA was synthesized using the Superscript III First-strand synthesis system (Invitrogen) and quantified using the Agilent Bioanalyzer. Bulk RNA-seq analysis was performed using the standard pipeline. In brief, pair-end RNA-seq reads were aligned using STAT/2.5.1b to the human reference genome hg38. Gene expression was measured using RESM/1.2/25 with expression values normalized into FPKM.

### Single-cell data analysis

For all single-cell data analysis, low-quality cells were filtered out and the same normalization was performed to eliminate cell-specific biases. For each cell, we calculated three quality control metrics: the number of expressed genes, the total number of transcripts and the proportion of transcripts in mitochondrial genes. The single-cell data matrix was column-normalized (divided by the total number of transcripts and multiplied by 10,000) and then log-transformed with pseudo-count +1.

For single-cell RNA-seq data on bulge SCs, cells from P30 WT, P56 WT and P56 *Tyr-Nras*^*Q61K*^ samples were combined, and the expression of genes with multiple Ensembl IDs was averaged. For quality control, cells with the total number of TPM counts of less than 750,000, with the proportion of TPM counts in mitochondrial genes of more than 20% and with the number of expressed genes of more than 7,000 or less than 2,000 were removed. In summary, 20 cells were removed, leading to 256 cells for downstream analyses. Clustering of cells was performed using the Seurat R package (V2.3). Principle component analysis (PCA) was first performed using highly variable genes, which were identified with an average expression of more than 0.01 and dispersion of more than 1. We regressed out the effects of the total number of transcripts and the transcripts in mitochondrial genes. The top 17 PCs were selected based on the Jackstraw method (JackStraw function). Using these top PCs, the Louvain modularity-based community detection algorithm was used to obtain cell clusters with resolution being 1.1, giving five clusters. The likelihood-ratio test was used to perform differential gene expression analysis between the clusters. Genes with a *P* value of less than 0.01 and a log fold change greater than 0.25 were considered as differentially expressed. To visualize cells onto a two-dimensional space, we performed *t*-distributed stochastic neighbour embedding. The relatedness of cell clusters was determined by performing unsupervised hierarchical clustering of average gene expression of cell clusters using the highly variable genes (correlation distance metric and average linkage). To determine the cell cycle phase of each cell, we used cell cycle-related genes, including a core set of 43 G1/S and 54 G2/M genes. For each cell, a cell cycle phase (G1, S and G2/M) was assigned based on its expression of these cell cycle-related genes using the CellCycleScoring function in Seurat.

### Statistics and reproducibility

Sample size calculations were not performed for mouse experiments, but *n* = 3 is a standard minimal sample size that in our previous studies was found to be sufficient to assess changes in hair growth in mice. Group sizes in animal experiments were derived from the power analysis performed on preliminary experimental data. Animals of both sexes were used, and analyses were not segregated by sex. Age of animals is defined in all experiments in postnatal days. Statistical analyses were performed using unpaired one-tailed or two-tailed (defined in the figure legends) Student’s *t*-tests. In all bar charts shown in figures, error bars are mean ± s.d. Statistical significance degree in figures is defined as follows: *P* ≥ 0.05 (not significant), *P ≤ 0.05 and **P ≤ 0.01; exact *P* values are provided in the figure legends. Differentially expressed gene analysis on RNA-seq data, reported in Supplementary Tables 1, 3, 4 and 5, was done using the edgeR package. When comparing gene expression between groups, the exact test (exactTest() function, two-sided) was performed for *P* value calculation after the negative binomial models were fitted and dispersion was calculated. *P* values were adjusted by using Benjamini and Hochberg’s approach for false discovery rate output. For gene ontology terms reported in Supplementary Tables 1, 3, 4 and 5, analysis was done using Metascape. *P* values were calculated using hypergeometric test, and then adjusted by using Benjamini and Hochberg correction. Exact *P* values are reported in the above-mentioned tables. All experiments were repeated independently with similar results of three times or more, and data shown in the figures are from representative experiments. The number of independent repeats for the representative experiments shown as micrographs are as follows: Fig. 1c (*n* = 3), Fig. 1d (*n* = 5), Fig. 1e (*n* = 3), Fig. 1k (*n* = 5), Fig. 2j (*n* = 3), Fig. 3g (*n* = 5), Fig. 4a–d (*n* = 3 each), Fig. 5d–h (*n* = 3 each), Extended Data Fig. 1a,b,d,f–h (*n* = 3 each), Extended Data Fig. 1c (*n* = 6), Extended Data Fig. 1e (*n* = 7), Extended Data Fig. 1j,k (*n* = 3 each), Extended Data Fig. 2a (*n* = 4), Extended Data Fig. 2d (*n* = 4), Extended Data Fig. 3j (*n* = 4), Extended Data Fig. 3k (*n* = 4), Extended Data Fig. 4a (*n* = 5), Extended Data Fig. 4l (*n* = 3), Extended Data Fig. 5a (*n* = 6), Extended Data Fig. 5f–j (*n* = 3 each), Extended Data Fig. 6a,b (*n* = 3 each), Extended Data Fig. 7i (*n* = 3), Extended Data Fig. 8a–c (*n* = 4 each), Extended Data Fig. 8d (*n* = 3), Extended Data Fig. 8e (*n* = 5), Extended Data Fig. 8f (*n* = 5), Extended Data Fig. 8g (*n* = 4), Extended Data Fig. 9e (*n* = 3), Extended Data Fig. 9l,m (*n* = 3 each). Experiments were not randomized or performed in a blinded manner, except where noted.

### Schematics

Schematics were prepared using Adobe Illustrator.

### Reporting summary

Further information on research design is available in the Nature Portfolio Reporting Summary linked to this article.

## Online content

Any methods, additional references, Nature Portfolio reporting summaries, source data, extended data, supplementary information, acknowledgements, peer review information; details of author contributions and competing interests; and statements of data and code availability are available at 10.1038/s41586-023-06172-8.

## Supplementary information

Supplementary Information This file contains Supplementary Discussion, Supplementary Figures 1 &2 and Supplementary References

Reporting Summary

Supplementary Table 1 Differential gene expression between sorted bulge stem cells in *Tyr-Nras*^*Q61K*^ mutant *vs*. control mice as revealed by bulk RNA-sequencing.

Supplementary Table 2 Single-cell RNA-sequencing gene expression matrix for sorted bulge stem cells from *Tyr-Nras*^*Q61K*^ mutant mice at postnatal day P56 and control mice at postnatal days P30 and P56.

Supplementary Table 3 Differential gene expression between bulge stem cell clusters as revealed by combined single-cell RNA-sequencing analysis of *Tyr-Nras*^*Q61K*^ mutant and control mouse cells.

Supplementary Table 4 Differential gene expression between sorted dermal melanocytes in *Tyr-Nras*^*Q61K*^ mutant *vs*. control mice as revealed by bulk RNA-sequencing.

Supplementary Table 5 Differential gene expression between nevus *vs*. non-nevus control human skin as revealed by bulk RNA-sequencing.

Supplementary Table 6 Sequence of mouse-specific and human-specific primers used for RT-PCR.

## Data Availability

Mouse bulk RNA-seq data are located at GSE111999, mouse single-cell RNA-seq data are located at GSE112722 and human bulk RNA-seq data are located at GSE112219. Processed bulk RNA-seq and single-cell data are provided in Supplementary Tables 1–5. Primer sequences are provided in Supplementary Table 6. Source data behind all graphs in main and extended data figures are provided with this paper. Full versions of all gels and blots are provided in Supplementary Fig. 1. Sequential gating strategies are provided in Supplementary Fig. 2. Source data are provided with this paper.

## Source data

Source Data Fig. 1
Source Data Fig. 2
Source Data Fig. 3
Source Data Fig. 4
Source Data Fig. 5
Source Data Extended Data Fig. 2
Source Data Extended Data Fig. 3
Source Data Extended Data Fig. 4
Source Data Extended Data Fig. 5
Source Data Extended Data Fig. 7
Source Data Extended Data Fig. 9

## Code availability

No custom code was used. Source data are provided with this paper.
