# Supplementary material for: Signalling by senescent melanocytes hyperactivates hair growth
Source: Nature. 2023 Jun 21;618(7966):808–17. doi: 10.1038/s41586-023-06172-8 (PMC10284692; doi:10.1038/s41586-023-06172-8)
Supplement: Supplementary file 1 — This file contains Supplementary Discussion, Supplementary Figures 1 &2 and Supplementary References [file 41586_2023_6172_MOESM1_ESM.docx]

**Supplementary Information**

**Supplementary Discussion 1: Factors determining tissue-level effects of senescent cells.** What determines if senescent cells will exert a promoting rather than deleterious effect on tissue growth and what do we learn in this regard from the hairy nevus model? First, we posit that preservation of intact tissue-resident SCs is a prerequisite for the growth-promoting effect of senescent cells. Indeed, we show that epithelial HF SCs, whose activity sustains hair growth, remain largely preserved in nevus skin and are thus available to respond to potential triggering signalling factors part of the SASP. Second, most SASP factors are short range-acting signalling molecules, and to be effective at stimulating tissue-resident SCs, senescent cells need to reside within an effective morphogenetic gradient range (no more than several hundred micrometers). Indeed, we observe that senescent dermal melanocytes in nevus skin are commonly positioned close to SC compartments of HFs. Third, for senescent cells to elicit effective signalling responses on target cells, their numbers and density likely need to be above a certain threshold. The OIS mechanism which includes transient pre-senescent expansion phase, is well suited for generating spatially-restricted dense clusters of senescent cells, as observed in the case of dermal senescent melanocyte clusters in nevus skin. Fourth, the molecular composition of SASP should include at least one ligand for which SCs express a cognate receptor, whose triggering elicits a SC activation program. We show that dermal senescent melanocytes express SPP1 and epithelial HF SCs express its receptor CD44.

**Supplementary Discussion 2: Molecular heterogeneity of SASP.** The molecular composition of SASP likely varies between different senescent cells, and factors affecting it can include the mode of senescence – OIS *vs.* DNA damage-induced *vs.* replicative *vs.* programmed senescence, as well as the original lineage of senescent cells – epigenetic profile of the original cell type can place restrictions on the genes available for expression by their senescent counterparts. Indeed, existing molecular evidence supports that lead SASP factors are heterogenous *in vivo* across senescent cell types. “Developmental” senescent cells that form widespread across mouse embryonic structures, including limb bud, tail tip, closing neural tube, fusing sternum, etc., express morphogenetic factors such as BMP7, FGF4 and WNT5A[^1^](#_ENREF_1)^,^[^2^](#_ENREF_2). Dominant SASP factors produced by senescent cells in adult healing tissues can include: IL-33 and CCN1 in senescent fibro-adipogenic cells of repairing skeletal muscle[^3^](#_ENREF_3)^,^[^4^](#_ENREF_4), IL-6 and CXCL1/2/5 in senescent stellate cells of regenerating liver[^5^](#_ENREF_5), and PDGF-AA in senescent fibroblasts and senescent endothelial cells in healing skin wounds[^6^](#_ENREF_6). Examples of lead SASP factors produced by cancer-associated senescent cells include: IL-6, IL-8 and TGFβ in senescent hepatic stellate cells upon hepatocellular carcinoma[^7^](#_ENREF_7)^,^[^8^](#_ENREF_8), IL-6 in senescent cancer associated fibroblasts (CAFs) from gastric cancer metastases[^9^](#_ENREF_9), IL-8 in senescent CAFs from pancreatic cancer[^10^](#_ENREF_10), and amphiregulin (AREG) in senescent stromal cells of prostate cancer[^11^](#_ENREF_11).

**
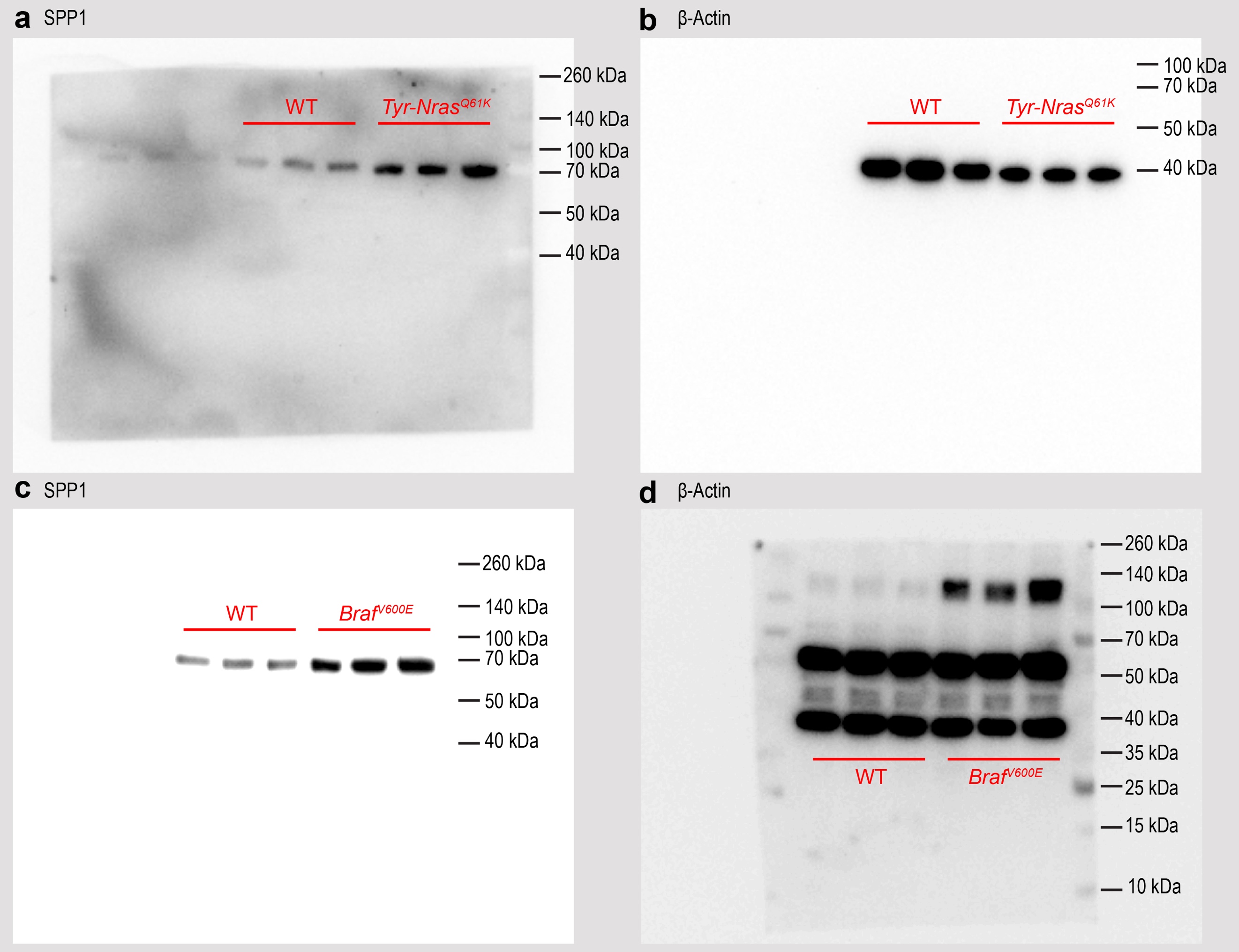
**

**Supplementary Fig. 1. Uncropped western blots from main Fig. 3. a,** Western blot for Fig. 3c showing SPP1 in three WT and three *Tyr-Nras^Q61K^* melanocyte samples. **b,** Western blot for Fig. 3c showing β-Actin in three WT and three *Tyr-Nras^Q61K^* melanocyte samples. **c,** Western blot for Fig. 3f showing SPP1 in three WT and three *Tyr-CreER^T2^;Braf^V600E^* melanocyte samples. **d,** Western blot for Fig. 3f showing β-Actin in three WT and three *Tyr-CreER^T2^;Braf^V600E^* melanocyte samples. Grey background is added to help differentiate the boundaries of the western blot images.

**
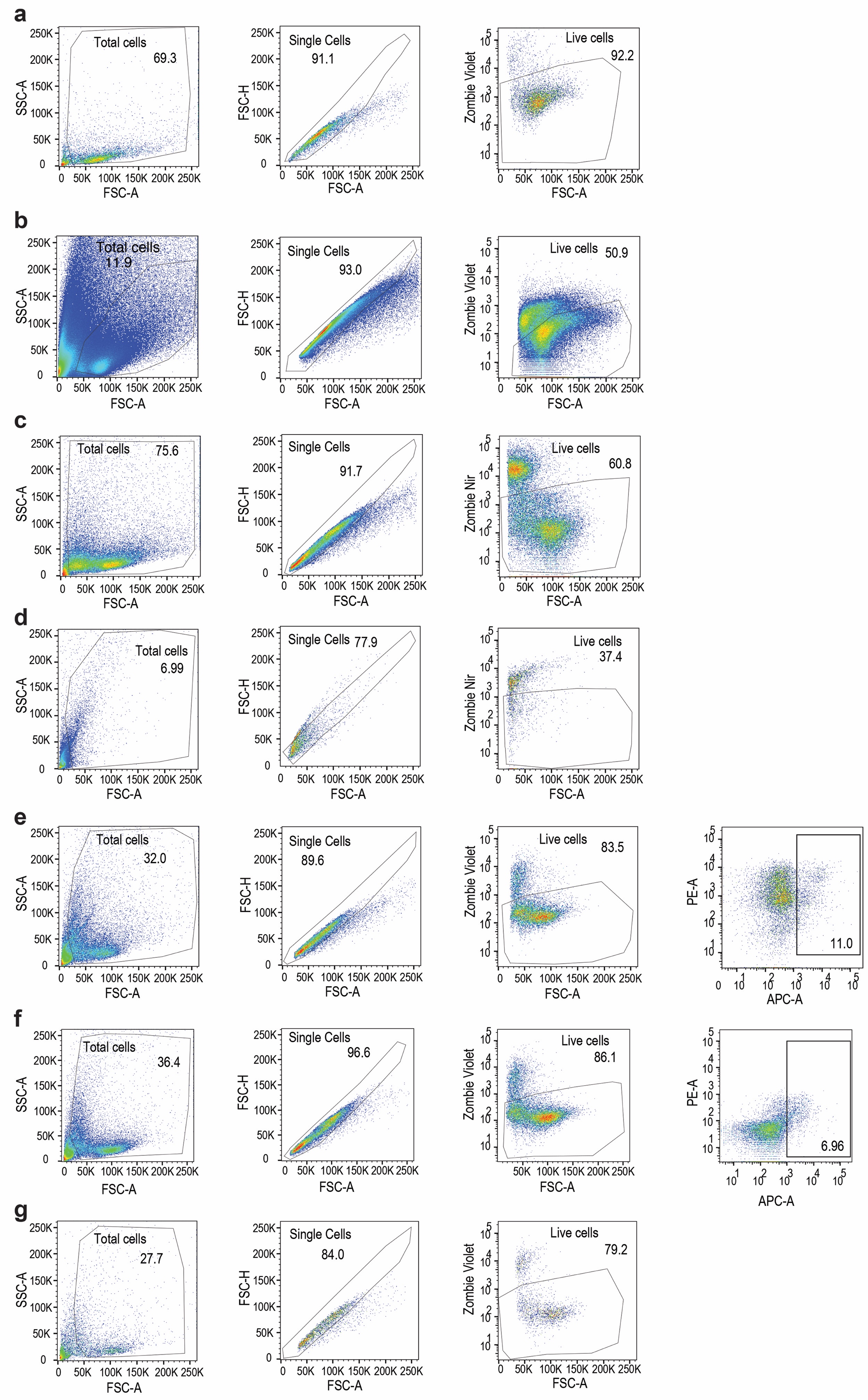
**

**Supplementary Fig. 2. Flow cytometry sequential gating strategies. a,** Gating strategy for main Fig. 1h and Extended Data Fig. 2b, 2c. **b,** Gating strategy for Extended Data Fig. 3a, 3c, 3f, 3h. **c,** Gating strategy for main Fig. 2i and Extended Data Fig. 5d, 7a, 9f, 9i. **d,** Gating strategy for main Fig. 2i and Extended Data Fig. 7b, 9g, 9j. **e,** Gating strategy for main Fig. 3a, 3d and Extended Data Fig. 4f, 4j. **f,** Gating strategy for main Fig. 3a, 3d and Extended Data Fig. 4g, 4k. **g,** Gating strategy for Extended Data Fig. 5c. Briefly, cell debris located in the bottom left corner of FSC (forward scatter) *vs.* SSC (side scatter) density plot were excluded by size and granularity (first column). Next, single cells were gated on the diagonal display
in FSC-H (forward scatter height) *vs.* FSC-A (forward scatter area) plot (doublet exclusion). Single live cells were then gated based on viability dye (Zombie Violet or Zombie Nir). Distinguishable negative population was selected as live cells. These single live cells were then gated based on staining patterns with antibodies indicated in main and Extended Data figures.

**Supplementary References**

1 Munoz-Espin, D. *et al.* Programmed cell senescence during mammalian embryonic development. *Cell* **155**, 1104-1118, doi:10.1016/j.cell.2013.10.019 (2013).

2 Storer, M. *et al.* Senescence is a developmental mechanism that contributes to embryonic growth and patterning. *Cell* **155**, 1119-1130, doi:10.1016/j.cell.2013.10.041 (2013).

3 Young, L. V. *et al.* Muscle injury induces a transient senescence-like state that is required for myofiber growth during muscle regeneration. *FASEB J* **36**, e22587, doi:10.1096/fj.202200289RR (2022).

4 Saito, Y., Chikenji, T. S., Matsumura, T., Nakano, M. & Fujimiya, M. Exercise enhances skeletal muscle regeneration by promoting senescence in fibro-adipogenic progenitors. *Nat Commun* **11**, 889, doi:10.1038/s41467-020-14734-x (2020).

5 Cheng, N., Kim, K. H. & Lau, L. F. Senescent hepatic stellate cells promote liver regeneration through IL-6 and ligands of CXCR2. *JCI Insight* **7**, doi:10.1172/jci.insight.158207 (2022).

6 Demaria, M. *et al.* An essential role for senescent cells in optimal wound healing through secretion of PDGF-AA. *Dev Cell* **31**, 722-733, doi:10.1016/j.devcel.2014.11.012 (2014).

7 Yoshimoto, S. *et al.* Obesity-induced gut microbial metabolite promotes liver cancer through senescence secretome. *Nature* **499**, 97-101, doi:10.1038/nature12347 (2013).

8 Nguyen, P. T. *et al.* Senescent hepatic stellate cells caused by deoxycholic acid modulates malignant behavior of hepatocellular carcinoma. *J Cancer Res Clin Oncol* **146**, 3255-3268, doi:10.1007/s00432-020-03374-9 (2020).

9 Yasuda, T. *et al.* Inflammation-driven senescence-associated secretory phenotype in cancer-associated fibroblasts enhances peritoneal dissemination. *Cell Rep* **34**, 108779, doi:10.1016/j.celrep.2021.108779 (2021).

10 Wang, T. *et al.* Senescent Carcinoma-Associated Fibroblasts Upregulate IL8 to Enhance Prometastatic Phenotypes. *Mol Cancer Res* **15**, 3-14, doi:10.1158/1541-7786.MCR-16-0192 (2017).

11 Xu, Q. *et al.* Targeting amphiregulin (AREG) derived from senescent stromal cells diminishes cancer resistance and averts programmed cell death 1 ligand (PD-L1)-mediated immunosuppression. *Aging Cell* **18**, e13027, doi:10.1111/acel.13027 (2019).
