# Supplementary material for: Signalling by senescent melanocytes hyperactivates hair growth
Source: Nature. 2023 Jun 21;618(7966):808–17. doi: 10.1038/s41586-023-06172-8 (PMC10284692; doi:10.1038/s41586-023-06172-8)
Supplement: Supplementary file 2 — Reporting Summary [file 41586_2023_6172_MOESM2_ESM.pdf]

nature portfolio

Corresponding author(s): Maksim V. Plikus, Xiaojie Wang

Last updated by author(s): Mar 22, 2023

Reporting Summary

Nature Portfolio wishes to improve the reproducibility of the work that we publish. This form provides structure for consistency and transparency in reporting. For further information on Nature Portfolio policies, see our Editorial Policies and the Editorial Policy Checklist.

Statistics

For all statistical analyses, confirm that the following items are present in the figure legend, table legend, main text, or Methods section.

n/a	Confirmed	
☐	☒	The exact sample size (*n*) for each experimental group/condition, given as a discrete number and unit of measurement
☐	☒	A statement on whether measurements were taken from distinct samples or whether the same sample was measured repeatedly
☐	☒	The statistical test(s) used AND whether they are one- or two-sided<br>*Only common tests should be described solely by name; describe more complex techniques in the Methods section.*
☒	☐	A description of all covariates tested
☐	☒	A description of any assumptions or corrections, such as tests of normality and adjustment for multiple comparisons
☐	☒	A full description of the statistical parameters including central tendency (e.g. means) or other basic estimates (e.g. regression coefficient) AND variation (e.g. standard deviation) or associated estimates of uncertainty (e.g. confidence intervals)
☐	☒	For null hypothesis testing, the test statistic (e.g. *F*, *t*, *r*) with confidence intervals, effect sizes, degrees of freedom and *P* value noted<br>*Give P values as exact values whenever suitable.*
☒	☐	For Bayesian analysis, information on the choice of priors and Markov chain Monte Carlo settings
☒	☐	For hierarchical and complex designs, identification of the appropriate level for tests and full reporting of outcomes
☒	☐	Estimates of effect sizes (e.g. Cohen's *d*, Pearson's *r*), indicating how they were calculated

Our web collection on statistics for biologists contains articles on many of the points above.

Software and code

Policy information about availability of computer code

Data collection	For bulk RNA-sequencing on mouse skin cells, total RNA was extracted from FACS sorted cells in biological triplicates with RNA integrity number (RIN) > 9.1 and 1ng of mRNA was used for full length cDNA synthesis, followed by PCR amplification using Smart-seq2. The libraries were sequenced on the Illumina Next-Seq500 system to an average depth of 10-30 million reads per library using paired 43bp reads.<br><br>For single-cell RNA-sequencing (scRNA-seq) on mouse skin cells, sorted cells were captured using the Fluidigm C1 chips as per manufacturer's protocol. A concentration of 200,000–350,000 cells per mL was used for chip loading. After cell capture, chips were examined visually under the microscope to determine the capture rate and empty chambers or chambers with multiple cells were excluded from the analysis. cDNA was synthesized and amplified on Fluidigm C1 Single-Cell Auto Prep System with Clontech SMARTer Ultra Low RNA kit and ADVANTAGE-2 PCR kit (Clontech). Single-cell RNA-sequencing libraries were constructed in 96-well plates according to Fluidigm C1 manual. Multiplexed libraries were analyzed on Agilent 2100 Bioanalyzer for fragment distribution and quantified using Kapa Biosystem's universal library quantification kit. Libraries were sequenced as 75bp paired-end reads on the Illumina Next-Seq500 platform.<br><br>For bulk RNA-sequencing on human skin tissue, RNA was extracted from human hairy nevus skin as well as normal skin from nevus edge using QIAGEN RNA extraction kit. cDNA was synthesized using Superscript III First-strand synthesis system (Invitrogen) and quantified using Agilent Bioanalyzer.
Data analysis	For both bulk and single-cell RNA-sequencing on mouse samples, reads were first aligned using STAR v.2.4.2a with parameters '--outFilterMismatchNmax 10 --outFilterMismatchNoverReadLmax 0.07 --outFilterMultimapNmax 10' to the reference mouse genome (mm10/genocode,vM8). Gene expression levels were quantified using RSEM v.1.2.25 with expression values normalized into Fragments Per Kilobase of transcript per Million mapped reads (FPKM). Samples with >1,000,000 uniquely mapped reads and >60% uniquely mapping efficiency were used for downstream analyses. Differential expression analysis was performed using edgeR v.3.2.2 on protein-coding genes and lncRNAs.

Differentially expressed genes were selected by using fold change (FC)≥2, false discovery rate (FDR)<0.05 and counts per million reads (CPM)≥2.

For all single-cell data analysis on mouse cells, low-quality cells were filtered out and the same normalization was performed to eliminate cell-specific biases. For each cell, we calculated three quality control metrics: the number of expressed genes, the total number of transcripts and the proportion of transcripts in mitochondrial genes. Single cell data matrix was column-normalized (divided by the total number of transcripts and multiplied by 10,000) and then log-transformed with pseudo-count +1.

For single-cell RNA-sequencing data on mouse bulge SCs, cells from P30 wild type, P56 wild type and P56 Tyr-NrasQ61K samples were combined, and the expression of genes with multiple Ensembl ID was averaged. For quality control, cells with the total number of TPM counts < 750,000, with the proportion of TPM counts in mitochondrial genes > 20%, and with the number of expressed genes > 7000 or < 2000 were removed. In sum, 20 cells were removed, leading to 256 cells for downstream analyses. Clustering of cells was performed using the Seurat R package (V2.3). Principle component analysis (PCA) was first performed using highly variable genes, which were identified with an average expression > 0.01 and dispersion > 1. We regressed out the effects of the total number of transcripts and the transcripts in mitochondrial genes. The top 17 PCs we selected based on the Jackstraw method (JackStraw function). Using these top PCs, the Louvain modularity-based community detection algorithm was used to obtain cell clusters with resolution being 1.1, giving five clusters. The likelihood-ratio test was used to perform differential gene expression analysis between the clusters. Genes with p-value less than 0.01 and log fold-change greater than 0.25 were considered as differentially expressed. To visualize cells onto a two-dimensional space, we performed t-distributed stochastic neighbor embedding (t-SNE). The relatedness of cell clusters was determined by performing unsupervised hierarchical clustering of average gene expression of cell clusters using the highly variable genes (correlation distance metric, average linkage). To determine the cell cycle phase of each cell, we used cell cycle-related genes, including a core set of 43 G1/S and 54 G2/M genes. For each cell, a cell cycle phase (G1, S, G2/M) was assigned based on its expression of these cell cycle-related genes using the CellCycleScoring function in Seurat.

Bulk RNA-sequencing analysis on human skin data was performed using standard pipeline. Briefly, pair-end RNA-sequencing reads were aligned using STAT/2.5.1b to the human reference genome hg38. Gene expression was measured using RESM/1.2/25 with expression values normalized into FPKM.

For flow cytometry and fluorescence-activated cell sorting (FACS) procedures, cells were sorted on FACSAria II sorters (BD Biosciences), while flow cytometry analysis was performed on LSRII flow cytometer (BD Biosciences). Data was analyzed with FlowJo software v.10.8.0.

For manuscripts utilizing custom algorithms or software that are central to the research but not yet described in published literature, software must be made available to editors and reviewers. We strongly encourage code deposition in a community repository (e.g. GitHub). See the Nature Portfolio guidelines for submitting code & software for further information.

Data

Policy information about availability of data

All manuscripts must include a data availability statement. This statement should provide the following information, where applicable:

- Accession codes, unique identifiers, or web links for publicly available datasets
- A description of any restrictions on data availability
- For clinical datasets or third party data, please ensure that the statement adheres to our policy

Mouse bulk RNA-seq data is located at GSE111999; mouse single-cell RNA-seq data is located at GSE112722; human bulk RNA-seq data is located at GSE112219. Processed bulk RNA-seq and single-cell data is provided in SI Tables 1 through 5. Primer sequences are provided in SI Table 6. Source data behind all graphs in main and extended data figures are provided with this paper. Full versions of all gels and blots are provided in SI Fig. 1. Sequential gating strategies are provided in SI Fig. 2.

Human research participants

Policy information about studies involving human research participants and Sex and Gender in Research.

Reporting on sex and gender

Disaggregated analysis by sex has not been performed, because there are no known differences in the hair-bearing status of melanocytic nevi in people.

Population characteristics

Surgical discard skin samples were collected for histological, immunohistological, bulk RNA-seq and transplantation studies.
Inclusion criteria:
1) Gender: female and male.
2) Age: 18-40 years old.
3) Region: scalp, face, neck, chest.

Exclusion criteria:
1) Previous surgical treatment.
2) Systemic treatment within 6 months.
3) Any other systemic disease, including diabetes and hypertension.

Recruitment

Subjects were recruited into the study via referral by physicians at Central South University (Changsha, China), National Taiwan University (Taipei, Taiwan), and/or Kyungpook National University (Daegu, Korea). An IRB-approved recruitment letter was provided to patients. Physicians screened patients' medical records to determine subject eligibility before approaching patients about study participation. Written (signed) informed consent was obtained from all subjects. Subjects were allowed to take home the unsigned consent form for review prior to signing it. All patients fitting the inclusion criteria were offered to participate in the study. Authors are not aware of the self-selection bias.

Ethics oversight

Collection of human skin samples was performed in compliance with relevant ethical regulations and was approved by the National Taiwan University Hospital (Taipei, Taiwan) and/or by the Kyungpook National University Hospital (Daegu, Korea) and/or by the Ethics Committee of Xiangya Hospital, Central South University (Changsha, China) and comply with guidelines from the Ministry of Science and Technology (MOST) of the People's Republic of China. Participants provided written informed consent. No identifiable images of human research participants are shown.

Note that full information on the approval of the study protocol must also be provided in the manuscript.

Field-specific reporting

Please select the one below that is the best fit for your research. If you are not sure, read the appropriate sections before making your selection.

☒ Life sciences ☐ Behavioural & social sciences ☐ Ecological, evolutionary & environmental sciences

For a reference copy of the document with all sections, see nature.com/documents/nr-reporting-summary-flat.pdf

Life sciences study design

All studies must disclose on these points even when the disclosure is negative.

Sample size

No sample size calculations were performed for mouse experiments. Skin of one mouse was used per one bulk RNA-seq or single-cell RNA-seq experiment. One human skin tissue sample was used per one bulk RNA-seq experiment. For immunohistochemical analyses, we used 3 or more mice as biological replicates. For all functional mouse experiments, 3 or more mice were used per experiment. N = 3 is a standard minimal sample size that in our previous studies was found to be sufficient to assess changes in hair growth in mice.

Data exclusions

For full length single-cell RNA-seq, cells with the total number of TPM counts < 750,000, with the proportion of TPM counts in mitochondrial genes > 20%, and with the number of expressed genes > 7000 or < 2000 were removed.

Replication

All key findings throughout the manuscript were independently replicated. In single-cell RNA-seq experiments, individual sequenced cells were considered as replicates. In all in vivo mouse experiments, at least 3 mice were used per experiment. In epithelial cell clonogenic assays, Western blot assays and FACS/cytometry assays biological replicates were used. Exact numbers of independent replicates for experiments are indicated either in the main and Extended Data figure legends or in the "Statistics and reproducibility" section of the Methods.

Randomization

Littermate mice were assigned into groups on the basis of genotype. For mouse experiments performed in animals of the same genotype, mouse inclusion into each experimental group was randomized.

Blinding

Single-cell RNA-seq analyses were unbiased. All cells were analyzed using computational algorithms that were not biased to recognize any particular cell types. In all functional transgenic mouse experiments, intra-dermal protein injection experiments, intra-dermal cell injection experiments and in skin wounding experiments, hair follicle regeneration phenotype was quantified using blinded approach, by investigators who did not know the genotype or experimental condition. For all other experiments, data quantification was also performed using blinded approach, by investigators who did not know the experimental condition.

Reporting for specific materials, systems and methods

We require information from authors about some types of materials, experimental systems and methods used in many studies. Here, indicate whether each material, system or method listed is relevant to your study. If you are not sure if a list item applies to your research, read the appropriate section before selecting a response.

Materials & experimental systems

n/a	Involved in the study	
☐	☒	Antibodies
☐	☒	Eukaryotic cell lines
☒	☐	Palaeontology and archaeology
☐	☒	Animals and other organisms
☒	☐	Clinical data
☒	☐	Dual use research of concern

Methods

n/a	Involved in the study	
☒	☐	ChIP-seq
☐	☒	Flow cytometry
☒	☐	MRI-based neuroimaging

Antibodies

Antibodies used

Following primary antibodies were used for immunostaining:
Rabbit anti-γH2AX (Cell Signaling, catalog # 9718; concentration 1:300),
Rabbit anti-TRP2 (Abcam, # ab74073; 1:200),
Rabbit anti-TRP2 (Abcam, # ab103463; 1:200),
Mouse anti-PCNA (Abcam, #ab29; 1:1000),
Rat anti-CD34 (ThermoFisher, # 14-0341-82; 1:100),

Rabbit anti-SOX9 (Millipore, # AB5535; 1:200),
Goat anti- SPP1 (R&D, # AF808; 1:100),
Goat anti-SPP1 (R&D, # AF1433; 1:300),
Rabbit anti-KRT14 (Abcam, # ab119695; 1:2000),
Rabbit anti-CD44 (ThermoFisher, # PA5-94934; 1:100),
Rabbit anti-SOX10 (Abcam, # ab180862; 1:100),
Rabbit anti-KRT5 (Biolegend, # 905501; 1:1000),
Goat anti-Pcad (R&D Systems, # AF761; 1:200).

Following primary antibodies were used cell sorting:
Mouse anti-γH2AX (BD Biosciences, catalog # 564718; 1:100),
Mouse anti-TRP2 (Santa Cruz Biotechnology, # sc-74439 AF647; 1:50),
Rat anti-Ki67 (ThermoFisher, # 58-5698-82; 1:50),
Rat anti-CD117 (Biolegend, # 105812; 1:100),
Rat anti-CD45 (Biolegend, # 103108; 1:50),
Rat anti-CD34 (BD Biosciences, # 560230; 1:50),
Rat anti-CD49f (BD Biosciences, # 555736; 1:100),
Rabbit anti-SPP1 (ThermoFisher, # 702184; 1:100).

Following antibodies were used for western blot:
Goat anti-mouse SPP1 (R&D, # AF808; 1:100),
Rabbit anti-βActin (Cell Signaling, # 4967; 1:1000).

Following secondary antibodies were used:
Donkey anti-rat AF555 (Abcam, # ab150154; 1:1000),
Donkey Igg-PE (SouthernBiotech # 0175-09; 1:100),
Donkey anti-rabbit AF555 (ThermoFisher, # A31572; 1:1000),
Donkey anti-mouse AF555 (ThermoFisher, # A31570; 1:1000),
Donkey anti-rabbit AF488 (ThermoFisher, # A21206; 1:1000),
Donkey anti-goat AF488 (ThermoFisher, # A11055; 1:1000),
Goat anti-rat AF488 (ThermoFisher, # A11006; 1:1000),
Goat anti-rabbit AF488 (Cell Signaling, # 4412s; 1:1000),
Goat anti-mouse AF555 (Cell Signaling, # 4409s; 1:1000),
Goat anti-rabbit AF555 (Cell Signaling, # 4413s; 1:1000),
PE donkey anti-rabbit Ig (Biolegend, # 406421; 1:100).

Validation

Antibodies were not separately validated by the investigators. All antibodies used in this study we purchased from trusted commercial sources, that provide validation statements on their respective web pages. Use of antibodies followed manufacturer's recommended protocols.

Validation statements and sue recommendations can be found as follows:
Rabbit anti-γH2AX (Cell Signaling, catalog # 9718) has been cited by 1,844 peer-reviewed papers (per vendor's website) - https://www.cellsignal.com/products/primary-antibodies/phospho-histone-h2a-x-ser139-20e3-rabbit-mab/9718

Mouse anti-γH2AX (BD Biosciences, catalog # 564718) has been cited by 7 peer-reviewed papers (per vendor's website) - https://www.bdbiosciences.com/en-us/products/reagents/flow-cytometry-reagents/research-reagents/single-color-antibodies-ruo/percp-cy-5-5-mouse-anti-h2ax-ps139.564718

Rabbit anti-TRP2 (Abcam, # ab74073) has been cited by 41 peer-reviewed papers (per vendor's website) - https://www.abcam.com/products/primary-antibodies/trp2dct-antibody-ab74073.html

Rabbit anti-TRP2 (Abcam, # ab103463) has been cited by 2 peer-reviewed papers (per vendor's website; discontinued) - https://www.abcam.com/products/primary-antibodies/trp2dct-antibody-ab103463.html

Mouse anti-TRP2 (Santa Cruz Biotechnology, # sc-74439 AF647) has been cited by 39 peer-reviewed papers (per vendor's website) - https://www.scbt.com/p/trp2-antibody-c-9

Mouse anti-PCNA (Abcam, # ab29) has been cited by 517 peer-reviewed papers (per vendor's website) - https://www.abcam.com/products/primary-antibodies/pcna-antibody-pc10-ab29.html

Rat anti-CD34 (ThermoFisher, # 14-0341-82) has been cited by 151 peer-reviewed papers (per vendor's website) - https://www.thermofisher.com/antibody/product/CD34-Antibody-clone-RAM34-Monoclonal/14-0341-82

Rabbit anti-SOX9 (Millipore, # AB5535) has been cited by over 100 peer-reviewed papers (per vendor's website) - https://www.emdmillipore.com/US/en/product/Anti-Sox9-Antibody,MM_NF-AB5535

Goat anti- SPP1 (R&D, # AF808) has been cited by 111 peer-reviewed papers (per vendor's website) - https://www.rndsystems.com/products/mouse-osteopontin-opn-antibody_af808
6
Goat anti-SPP1 (R&D, # AF1433) has been cited by 26 peer-reviewed papers (per vendor's website) - https://www.rndsystems.com/products/human-osteopontin-opn-antibody_af1433

Rabbit anti-SPP1 (ThermoFisher, # 702184) has been cited by 1 peer-reviewed paper (per vendor's website) - https://www.thermofisher.com/antibody/product/OPN-R-Antibody-clone-24H5L3-Recombinant-Monoclonal/702184

Rabbit anti-KRT14 (Abcam, # ab119695) has been cited by 3 peer-reviewed papers (per vendor's website) - https://www.abcam.com/

products/primary-antibodies/cytokeratin-14-antibody-sp53-ab119695.html

Rabbit anti-CD44 (ThermoFisher, # PA5-94934) has been tested and demonstrated as specific by the vendor in HCT116 cells, HeLa cells, intestinal tissue, testis tissue, ovary tissue - https://www.thermofisher.com/antibody/product/CD44-Antibody-Polyclonal/PA5-94934

Rabbit anti-SOX10 (Abcam, # ab180862) has been cited by 7 peer-reviewed papers (per vendor's website) - https://www.abcam.com/products/primary-antibodies/sox10-antibody-epr4007-104-ab180862.html

Rabbit anti-KRT5 (Biolegend, # 905501) has been cited by 11 peer-reviewed papers (per vendor's website) - https://www.biolegend.com/en-us/products/keratin-5-polyclonal-antibody-purified-10956

Goat anti-Pcad (R&D Systems, # AF761) has been cited by 10 peer-reviewed papers (per vendor's website) - https://www.rndsystems.com/products/mouse-p-cadherin-antibody_af761

Rat anti-Ki67 (ThermoFisher, # 58-5698-82) has been cited by 63 peer-reviewed papers (per vendor's website) - https://www.thermofisher.com/antibody/product/Ki-67-Antibody-clone-SolA15-Monoclonal/58-5698-82

Rat anti-CD117 (Biolegend, # 105812) has been cited by 105 peer-reviewed papers (per vendor's website) - https://www.biolegend.com/fr-ch/products/apc-anti-mouse-cd117-c-kit-antibody-72

Rat anti-CD45 (Biolegend, # 103108) has been cited by 233 peer-reviewed papers (per vendor's website) - https://www.biolegend.com/en-us/products/fitc-anti-mouse-cd45-antibody-99

Rat anti-CD34 (BD Biosciences, # 560230) has been cited by 11 peer-reviewed papers (per vendor's website) - https://www.bdbiosciences.com/en-eu/products/reagents/flow-cytometry-reagents/research-reagents/single-color-antibodies-ruo/alexa-fluor-647-rat-anti-mouse-cd34.560230

Rat anti-CD49f (BD Biosciences, # 555736) has been cited by 3 peer-reviewed papers (per vendor's website) - https://www.bdbiosciences.com/en-us/products/reagents/flow-cytometry-reagents/research-reagents/single-color-antibodies-ruo/pe-rat-anti-human-cd49f.555736

Rabbit anti-βActin (Cell Signaling, # 4967) has been cited by 19,419 peer-reviewed papers (per vendor's website) - https://www.cellsignal.com/products/primary-antibodies/b-actin-antibody/4967

Eukaryotic cell lines

Policy information about cell lines and Sex and Gender in Research

Cell line source(s)	Primary adult mouse skin epithelial bulge and hair germ cells were assayed in vitro for clonogenic potential. Primary P0 mouse skin melanocytes were assayed in vitro in H2O2-induced senescence experiments.
Authentication	n/a
Mycoplasma contamination	n/a
Commonly misidentified lines (See ICLAC register)	n/a

Animals and other research organisms

Policy information about studies involving animals; ARRIVE guidelines recommended for reporting animal research, and Sex and Gender in Research

Laboratory animals	The following mouse lines were used: Tyr-NrasQ61K, Tyr-rtTA, Tyr-CreERT2, Tyr(C-2J), BrafV600E, p53flox, Spp1-/-, Spp1flox, tetO-Spp1, Cd44-/-, Cd44flox, K14-Cre, K14-CreERT, K14-H2B-GFP, K14-Edn3, K14-Kitl, tdTomato, TOPGAL, nude, SCID. Tissue-specific mouse models were produced by crossing either Cre- or CreER-carrying animals with flox-ed gene carrying animals, or rtTA-carrying animals with tetO-carrying animals. Ages of mice used in this study were between postnatal days P0 and P100. Mice were housed under temperature in the range of 21-23°C, relative humidity in the range of 35-50% and under 12 hours light / 12 hours dark cycle.
Wild animals	This study did not involve wild animals.
Reporting on sex	Both female and male mice were included in the study. Littermates of both sexes were randomly assigned to experimental groups. Data analysis was performed jointly on male and female mice. Formation of melanocytic nevi and hair growth in nevus skin occurs efficiently in animals of both sexes.
Field-collected samples	This study did not involve animals collected from the field.
Ethics oversight	Animal experiments were performed in compliance with relevant ethical regulations and were approved by animal research oversight committees at China Agricultural University (to Z.Y.) and/or Gifu University (to T.K.) and/or National Taiwan University (to C.-H.K.) and/or University of California, Irvine (to B.A. and/or A.K.G. and/or M.V.P.) and/or Central South University (to J.L.) and/or Kyungpook National University (to J.W.O.).

Note that full information on the approval of the study protocol must also be provided in the manuscript.

Flow Cytometry

Plots

Confirm that:

☒ The axis labels state the marker and fluorochrome used (e.g. CD4-FITC).

☒ The axis scales are clearly visible. Include numbers along axes only for bottom left plot of group (a 'group' is an analysis of identical markers).

☒ All plots are contour plots with outliers or pseudocolor plots.

☒ A numerical value for number of cells or percentage (with statistics) is provided.

Methodology

Sample preparation	Dorsal skin was digested into single cells with Dispase II solution (Roche), followed by Collagenase I solution (Life Technologies). Cells were filtered first through 70 µM and then 40 µM strainers. Viability dye (Biolegend) was used to exclude dead cells. Cell suspension was stained with primary antibodies in FACS staining buffer (1% BSA in PBS with 2 mM EDTA) for 30 minutes on ice before sorting.
Instrument	FACSAria II sorters (BD Biosciences), LSRII flow cytometer (BD Biosciences).
Software	FlowJo software (version 10.8.0).
Cell population abundance	The purity of sorted cells was >90% as tested by running post-sort samples on flow cytometer machine.
Gating strategy	Step 1: Cell debris that were located in the bottom left corner of FSC (forward scatter) vs. SSC (side scatter) density plot were excluded by size and granularity.<br>Step 2: Single cells were gated on a diagonal display in FSC-H (forward scatter height) vs. FSC-A (forward scatter area) plot.<br>Step 3: Single live cells were gated on the basis of Zombie violet viability dye staining. A distinguishable violet negative population was selected as live cells.<br>Step 4: Obtained single live cells were then gated as follows:<br>a) APC-TRP2 positive (x-axis) and PE-Ki67 negative (y-axis), or<br>b) APC-TRP2 positive (x-axis) and PerCP-Cy5.5-gH2AX positive (y-axis), or<br>c) APC-TRP2 positive (x-axis) and PE-SPP1 positive (y-axis), or<br>d) APC-TRP2 positive (x-axis) and PE-Annexin V positive (y-axis), or<br>e) APC-CD117 positive (x-axis) and FITC-CD45 negative (y-axis), or<br>f) Pacific Blue-CD49 positive (x-axis) and APC-34 positive (y-axis), and then as AF-488 Edu positive (x-axis), or<br>g) tdTomato positive (x-axis).

☒ Tick this box to confirm that a figure exemplifying the gating strategy is provided in the Supplementary Information.
